# Current Trends in Gelatin-Based Drug Delivery Systems

**DOI:** 10.3390/pharmaceutics15051499

**Published:** 2023-05-15

**Authors:** Francesca Milano, Annalia Masi, Marta Madaghiele, Alessandro Sannino, Luca Salvatore, Nunzia Gallo

**Affiliations:** 1 Department of Engineering for Innovation, University of Salento, Via Monteroni, 73100 Lecce, Italy; 2 Typeone Biomaterials Srl, Via Europa 113, 73021 Calimera, Italy

**Keywords:** gelatin, drug delivery systems, nanoparticles, microparticles, clinical studies

## Abstract

Gelatin is a highly versatile natural polymer, which is widely used in healthcare-related sectors due to its advantageous properties, such as biocompatibility, biodegradability, low-cost, and the availability of exposed chemical groups. In the biomedical field, gelatin is used also as a biomaterial for the development of drug delivery systems (DDSs) due to its applicability to several synthesis techniques. In this review, after a brief overview of its chemical and physical properties, the focus is placed on the commonly used techniques for the development of gelatin-based micro- or nano-sized DDSs. We highlight the potential of gelatin as a carrier of many types of bioactive compounds and its ability to tune and control select drugs’ release kinetics. The desolvation, nanoprecipitation, coacervation, emulsion, electrospray, and spray drying techniques are described from a methodological and mechanistic point of view, with a careful analysis of the effects of the main variable parameters on the DDSs’ properties. Lastly, the outcomes of preclinical and clinical studies involving gelatin-based DDSs are thoroughly discussed.

## 1. Introduction

Traditional forms of drug administration are often associated with side effects, mainly related to the non-specific biodistribution and uncontrolled concentration of the drug [1]. Moreover, they suffer from poor absorption at the target site, poor bioavailability, premature excretion from the body, and the requirement of multiple administrations [2]. Each drug has a therapeutic window, but for standard oral or injectable forms, the concentration of the pharmaceutical agent fluctuates between the minimum and maximum therapeutic range, leading to the loss of efficacy and toxicity. Due to all these reasons, controlled drug delivery systems (DDSs) are being designed and produced. Moreover, evidence for DDSs’ ability to increase drugs’ safety and efficacy has enhanced their development [3]. Thus, DDSs are historically defined as formulations or devices able to administrate therapeutic substances in a finely controlled manner [4]. These systems include the delivery of the therapeutic agent, its release, and its subsequent transport through the target site [4]. Accordingly, DDSs include micro- and nano-sized delivery systems, as well as 2D or 3D hydrogels. The historical evolution of DDSs linearly correlates with the decrease in the size of the final system. Starting from the 1960s up to the 1970s and 1980s, macroscopic DDSs were development, passing through the microscopic era between the 1970s and 1980s, up to the development of nanometric DDSs, which started during the 1970s and continues today [5]. Thus, while hydrogel-based DDSs were developed for the controlled and local delivery of drugs, micro- and nano-sized particles have also been developed for bioactive agents’ systemic or targeted delivery. DDSs have been developed in order to preserve the bioactive agents’ safety, to increase their efficacy, and to control their release kinetics to keep the concentration of the drug within the desired therapeutic range with a single application for a longer duration [6]. Consequently, other potential benefits that can be obtained from controlled DDSs include tissue-specific drug delivery, the reduction of the dosing frequency, the increase of the bioavailability of the drug via protection from the metabolism by enzymes or chemicals. All these advantages allow reducing the follow-up care and increasing patients’ comfort and compliance [6].

A wide variety of biodegradable polymers have been developed, investigated, and employed for the development of DDSs. Among them, collagen or its derivatives have attracted interest because of their intrinsic advantageous properties [7].

Collagen is a structural protein, which makes up about 25–35% of the total protein body mass. It is a ubiquitous protein, present in all vertebrates’ connective tissues, but it is especially expressed in the skin, tendons, and ligaments [8]. Collagen is a highly hierarchically organized protein. Its primary structure consists of the distinct and highly inter-species-conserved repetition of the (Gly-X-Y)n triplet, where “Gly” represents glycine, “X” usually represents lysine, and “Y” usually represents hydroxyproline. Thus, almost one third of the polypeptide chain of about 1000 amino acids is represented by glycine [9]. Each collagen monomeric unit consists of three right-handed polyproline-II helices (secondary structure), assembled to form a right-handed triple helix (tertiary structure). Gelatin derives from collagen’s partial acid-, alkaline-, or heat-based hydrolysis [10]. Therefore, its primary structure is almost the same as collagen.

Both collagen and gelatin-based DDSs have been developed for the delivery of various agents such as drugs, genes, growth factors, cells, and proteins [11,12,13,14,15,16,17,18,19,20,21,22,23,24,25,26,27]. Despite collagen having numerous advantages such as availability, biocompatibility, low immunogenicity, and biodegradability [28,29], gelatin is preferred as a biopolymer in designing micro- and nano-DDSs due to its additional advantages over collagen such as the ease of manufacturing and customization. Compared to collagen, gelatin is characterized by a higher number of accessible functional groups, which allow multiple coupling modifications with crosslinkers or targeted ligands, which are particularly useful in the development of targeted vehicles for drug delivery [24,30].

Among several types of gelatin-based DDSs, this review focuses only on microparticle and nanoparticle systems. After a brief overview of the properties of gelatin, a summary of the state-of-the-art of manufacturing techniques commonly used to obtain micro- and nano-sized gelatin-based DDSs, unloaded or loaded with model compounds or drugs is provided. Particular attention was paid to the parameters that influence each mentioned process, as well as their impact on the final materials. Lastly, the preclinical and clinical progress over the last twenty years is thoroughly discussed.

## 2. Methodology

The electronic search engines used in this research were PubMed (https://pubmed.ncbi.nlm.nih.gov, accessed on 1 February 2023), ScienceDirect (https://www.sciencedirect.com, accessed on 1 February 2023), Google scholar (https://scholar.google.com, accessed on 1 February 2023), and the U.S. National Library of Medicine (https://clinicaltrials.gov/, accessed on 1 February 2023). The keywords used were “gelatin”, “nanoparticles”, “microparticles”, and “drug delivery systems”. Several synonyms of these terms were used in the search for each component. The search included all studies related to gelatin-based DDSs, including preclinical and clinical trials, prospective case series, retrospective reviews, and case reports, independent of their level of evidence. A total of 45 preclinical and clinical studies (from 2000 to 2023) were screened and are discussed.

## 3. Gelatin: Structure and Properties

Gelatin is a natural biopolymer that is intrinsically biocompatible and biodegradable, has low immunogenicity, and is classified as Generally Recognized as Safe (GRAS) by the United States Food and Drug Administration (FDA) [31]. It consists of an amphoteric polymer that is derived from collagen by alkaline-, acid-, or heat-based hydrolysis [10]. These treatments cause the rupture of collagen’s structural organization, leading to the loss of its conformation to varying extents. Therefore, as for collagen, the extraction sources (i.e., equine, bovine, porcine, ovine, fish) and tissues (i.e., skin, tendon, scales, bone), and the animal age, besides the applied extraction procedures, represent all the parameters influencing gelatin’s properties [32]. Commercially, there are two types of gelatin, Type A and Type B. Type A cationic gelatin results from the partial acid hydrolysis of collagen. During this treatment, the amide groups of glutamine and asparagine are converted into carboxyl groups, resulting in the protein isoelectric point shift to higher values (pI = 7–9) [33]. Type B anionic gelatin derives from the alkali-based treatment of collagen. During the alkaline hydrolysis treatment, the partial removal of the asparagine and glutamine amide groups occurs, with a consequent increase in the aspartic and glutamic acid content [34,35]. The consequent increase of the carboxyl groups makes Type B gelatin negatively charged, with a lower isoelectric point (pI = 4.7–5.5) [32,36,37,38]. Accordingly, depending on the extraction process type and parameters, gelatin can have different isoelectric points, which depend on the degree of dissociation of its free carboxyl and amino groups [32,39].

**Figure 1:**
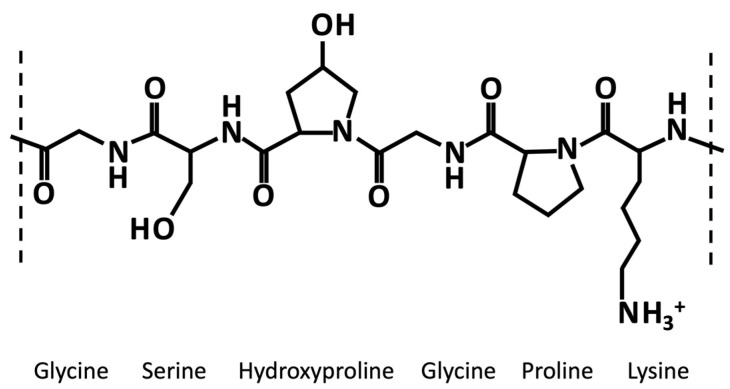
Exemplary chemical structure of a fragment of gelatin α-chain characterized by the repetition of the triplet (Gly-X-Y)n, where X and Y are usually proline an hydroxyproline, respectively.

Generally, like collagen, gelatin is characterized by the repetition of the triplet (Gly-X-Y)n (Figure 1). One third of the chain is made up of glycine, while another third is proline or hydroxyproline [40]. The hydroxylation of proline and lysine residues in 4-hydroxyproline and ε-hydroxylysine, respectively, is a posttranslational modification, which is present almost exclusively in collagen [41]. Gelatin owns cationic and anionic groups along with hydrophobic groups approximatively in a ratio of 1:1:1. Thus, about 13% of the polypeptide chain of gelatin consists of positively charged amino acid residues (i.e., mainly lysine and arginine residues), about 12% of negatively charged amino acid residues (i.e., mainly glutamic and aspartic acid), and about 11% of hydrophobic residues (i.e., leucine, isoleucine, methionine, and valine) [40].

**Figure 2:**
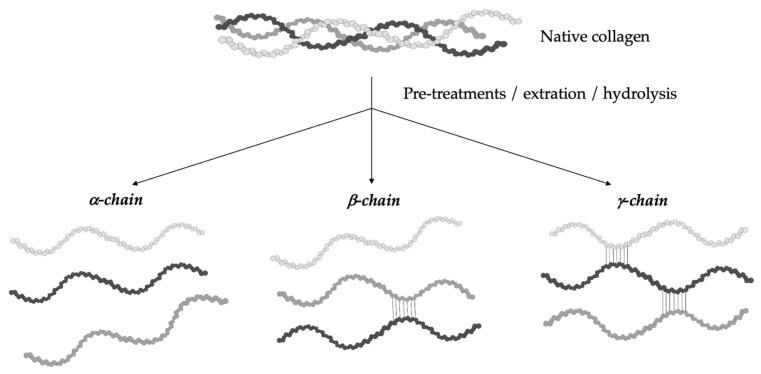
Gelatin is a heterogeneous mixture of water-soluble proteins with different types of chains and molecular weights depending on the production process.

The hydrolytic conversion of collagen breaks its polypeptide chains and natural structural organization. Therefore, gelatin cannot be considered as a single chemical entity with a precise molecular weight, but it rather consists of mixtures of polypeptide chains with different molecular weights that can fall in a specific range. Depending on the production process, gelatin can be characterized by different types of chains (with variable molecular weights). In particular, they could be found as (i) single α-chains of 80–125 kDa, (ii) two α-chains crosslinked in a covalent way (β-chains) of 160–250 kDa, or (iii) three covalently crosslinked α-chains (γ-chains) of 240–375 kDa (Figure 2) [39,42,43,44]. Gelatin’s protein pattern can be determined by several analytical techniques. Among them, electrophoresis and chromatography are the most-commonly performed [45].

**Figure 3:**
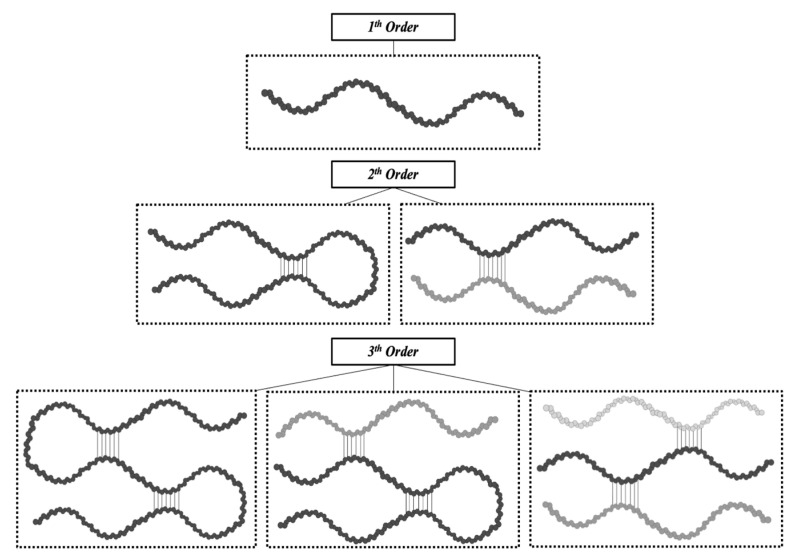
The different types of gelatin chain organization.

Consequently, it is clear how the conformational organization of gelatin differs from that of the highly organized native collagen depending on the denaturation degree of collagen. Advanced techniques can identify the extent of collagen denaturation. Indeed, X-ray diffraction has shown a certain degree of fibril-like structures in gelatin, although these structures are not comparable to the well-organized collagen networks [39]. Depending on the concentration of gelatin and the temperature and the energy required for the formation of secondary structures, the polypeptide chains of gelatin may have different spatial arrangements and, thus, different interactions during gelling [46]. As reported by Guo et al. [47], three different orders of organization can be found (Figure 3). The first order is characterized by single α-chains. In the second order, α-chains can form inter-chain or intra-chain interactions, creating a loop. Lastly, the third level of order can be constituted by three different α-chains, or two α-chains, one of which creates a loop, or a single α-chain with two loops. Single-looped helices can be found in diluted solutions, while non-looped helices are more commonly found in concentrated solutions.

Gelatin’s rheological properties (gel strength, viscosity) and thermal stability (melting and gelling temperature) define its quality, in addition to basic physico-chemical characteristics (solubility, composition, transparency, color, smell, and taste) [48]. Gel strength is defined by the so-called “Bloom value” [49,50,51], and it was found to decrease when the pH value was below 5 and above 9, while it remained almost constant in the pH range 5–9, with some variations [52]. Thus, the determination of the Bloom force is usually performed at pH values from 4.6 to 7.0 [49]. Another important physical property of gelatin is its viscosity, which depends on concentration, temperature, and pH. Indeed, viscosity was found to increase with polymer concentration and decrease with temperature and pH. Gelatin’s thermal stability is another important parameter that is influenced by several parameters such as polymer concentration, molecular weight distribution, and Bloom value [52]. However, gelatin’s melting point is usually found in the range 28–31 °C for mammal–derived gelatin and in the range of 11–28 °C for fish-derived gelatin [48,53]. Generally, the gel strength, gelling, and melting points of mammalian-derived gelatins have been revealed to be much higher than those of fish-derived gelatins. Indeed, the typical gel strength, gelling point, and melting point temperatures for mammalian gelatins are found in the range 100–300 Bloom, 20–25 °C and 28–31 °C, respectively, in comparison to fish gelatins’ values, which are about 70–270 Bloom, 8–25 °C and 11–28 °C, respectively [48,53]. As regards gelatin’s gelling time, its thermo-reversible gelation mechanism has been extensively studied. It is known that, at low temperatures, gelatin chains undergo a conformational disordered–ordered transition and are able to form thermo-reversible networks by the formation of hydrogen bonds [52,54]. In particular, gelatins are found in the sol state at high temperatures (>40 °C) as single coils. Above a determined critical concentration (usually about 1%), they are able to assemble into thermo-reversible gels with a disordered organization when the temperature is cooled down below 30 °C [55,56].

Thus, gelatin owns many advantages such as low cost, easy availability, biodegradability, and low immunogenicity, besides high biocompatibility and intrinsic bioactivity [40,57] thanks to the presence of specific arginine–glycine–aspartic (RGD) sequences, which are able to promote cell adhesion [24]. Moreover, being characterized by different functional groups that are easily accessible for chemical modifications (such as coupling with crosslinkers or target ligands), gelatin is widely used as a material for the manufacturing of substrates for a wide range of applications in the biomedical, pharmaceutical, cosmetic, and food sectors [58,59].

Indeed, gelatin is widely used in the food sector as a thickener (e.g., in sweets and jams), as a clarifying agent for drinks (e.g., wine, beer, fruits, and vegetables juices), an emulsifier (e.g., confectionery products), a stabilizer (e.g., ice creams, cream cheeses, and cottage cheese, as well as in food foams and salads), a texturizer, and a film former in coatings for meat and confectioneries [50,60,61,62].

As regards the cosmetic sector, gelatin is used as a gelling ingredient in various products (e.g., face creams, body lotions, shampoos, hairsprays, sunscreens, and bath salts) for its moisturizing action [63,64]. Moreover, its hydrolysates are used for nutricosmetic applications thanks to their antiaging effect [65].

Although gelatin seems to be mainly utilized in the food sector, actually, it is mostly used in the pharmaceutical sector as binder in the production of drugs [66], a stabilizer in vaccines [67], a material for the development of capsules and ointments [30], a matrix of implants and wound dressings [68], and for plasma expanders [69]. Lastly, in the biomedical field, gelatin is also used as a biomaterial for the development of DDSs and tissue engineering/regenerative medicine constructs [70]. In particular, gelatin has been revealed to be a good biomaterial for the manufacturing of DDSs thanks to its chemical versatility. Its high suitability in several synthesis techniques has pointed out its potential as a carrier of many types of bioactive compounds and its ability to tune and control the release kinetics of select drugs.

## 4. Gelatin-Based DDSs

**Figure 4:**
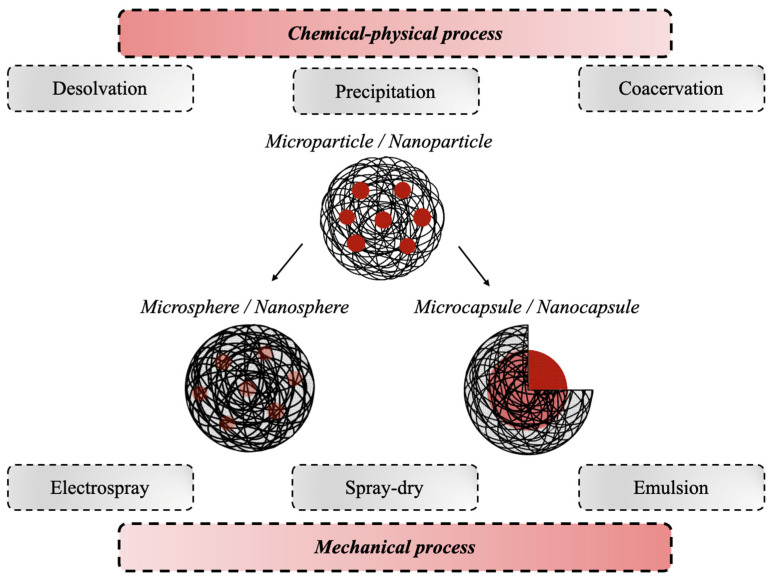
Schematic representation of the most-used production processes for the synthesis of gelatin-based micro- and nano-DDSs.

According to the 2011 IUPAC recommendations, the terms polymeric microparticles and nanoparticles are used to describe polymeric particles of any shape with a diameter of approximately 0.1–100 μm and 1–100 nm, respectively [71]. Both systems can be divided into two morphological classes, named spheres and capsules, respectively. The first ones are polymeric particles having a spherical shape, where the drug is physically and uniformly dispersed in the polymer matrix, while the second ones are polymeric particles comprising at least two-phase domains, the drug nucleus (fluid or solid), which can later be released, lying within a polymeric envelope that forms the outer layer [71]. Several techniques have been developed for the preparation of gelatin-based micro- and nano- DDSs [72,73,74], which can be divided into physico-chemical and mechanical processes. The physico-chemical processes are based on the precipitation or flocculation of the colloidal material and include desolvation, precipitation, and coacervation. The mechanical processes instead are based on the use of a specific type of equipment to produce particles, such as electrospray, spray drying, and emulsion (Figure 4) [75].

As shown by Table 1, in the synthesis of micrometer and nanometer devices, there are no limitations to the nature of the gelatin used, since many studies utilize both Type A and Type B to obtain DDSs with the desired release kinetics of biologically active molecules [76,77,78,79,80]. As regards the gelatin source, gelatin derived from porcine [81,82,83] and bovine skin [84,85] are the most-commonly used. Only a few works reported using gelatin derived from beef nails [79], camel skin [86], and fish skin [87] for the development of gelatin-based DDSs. However, many works did not report the animal source or the gelatin type, as indicated in Table 1 as “not defined or reported” (N. d.) information.

Different synthesis techniques usually have in common the use of chemical crosslinkers that are necessary to tune DDSs’ degradation and drug release kinetics. The most-used crosslinkers are glutaraldehyde (GA) [88,89,90], followed by formaldehyde (FA), genipin [91], dialdehyde carboxymethyl cellulose (DCMC) [92], methylenebisacrylamide (MBA) [93], formalin [94], diisopropylcarbodiimide (DIC) [95], and calcium chloride (CaCl_2_) [96,97]. Much less-common are the physical crosslinking by means of heat [98] and the enzymatic one, mediated by enzymes such as transglutaminase (TG) [99].

Gelatin-based DDSs have been revealed to be able to encapsule a huge variety of compounds, which goes from growth factors (e.g., transforming growth factor beta 1 (TGF-β1), basic fibroblast growth factor (bFGF), vascular endothelial growth factor (VEGF), bone morphogenetic protein 2 (BMP-2)) [27,76,91,100], vitamins (e.g., α-Tocopherol, vitamin D3) [83,99], and plant extracts (e.g., Phyllanthus urinaria extract, cocoa-derived polyphenolic extract, capsaicin, curcumin) [92,101,102,103] to cells (e.g., L929 fibroblasts, human bone marrow stromal cells, human adipose-derived stem cells) [93,96,97,104].

**Table 1:** Overview of the production methods of gelatin-based particles for the delivery of bioactive compounds.

DDS Synthesis Technique	DDS Type	Type of Gelatin	Gelatin Source	Encapsulated Bioactive Compound	Crosslinker	Ref.
Desolvation	Nanoparticles	Type A	Porcine skin	Ibuprofen sodium	CaCl_2_	[105]
				Didanosine	GA	[81]
				Moxifloxacin	GA	[106]
				Timol maleate	GA	[107]
				-	GA	[108]
		Type B	Bovine skin	Rutin	GA	[109]
				Rosiglitazone	GA	[84]
		Type A and B	Porcine skin and bovine skin	BMP-2, bFGF	GA	[100]
				Texas Red	GA	[76]
				Cardamom	GA	[77]
				Amphotericin B	GA	[78]
			Porcine skin and beef nails	Fluorescein-5-isothiocynate	GA	[79]
		N. d.	Bovine skin	Bovine serum albumin	GA	[110]
			Fish skin	-	GA	[111]
			Camel skin	-	GA	[86]
Nanoprecipitation	Nanoparticles	Type B	Bovine skin	Lysozyme	DIC	[95]
				-	–	[112]
				-	GA	[113]
				Fluorescein-5-isothiocynate	GA	[114]
				Dextran	GA	[70]
				Tizadine hydrochloride gatifloxacin	GA	[113]
				Metoprolol	GA	[90]
		N. d.	Porcine	Erythromycin	GA	[115]
			N. d.	Cocoa-derived polyphenolic extract	GA	[102]
		Type A	Porcine skin	Zaltoprofen	GA	[82]
		Type A and B	Porcine skin and bovine skin	Non-steroidal anti-inflammatory drugs	GA	[80]
Coacervation	Microcapsules/nanocapsules	Type A	Porcine skin	α-Tocopherol	GA	[83]
				Vitamin D3	TG	[99]
				Capsaicin	GA	[116]
		N. d.	N. d.	Capsaicin	GA	[103]
		N. d.	N. d.	Zeaxanthin	TG	[117]
		N. d.	N. d.	Phenacetin	Formalin	[94]
		N. d.	N. d.	Berberine hydrochloride Gallic acid	–	[118]
		Type B	Bovine skin	Geraniol oil	GA	[85]
		N. d.	N. d.	Moxa oil	FA	[119]
		N. d.	Fish	Fish oil	CaCl_2_	[120]
Emulsion	Microspheres/nanospheres	Type B	N. d.	Mitomycin C-dextran conjugate	FA	[121]
			Bovine skin	Sodium fluoride	GA	[88]
		Type A	Porcine skin	TGF-β1	Genepin	[27]
				L929 fibroblasts	MBA	[93]
		Type A and B	N. d.	bFGF	GA	[122]
			Porcine skin and bovine skin	BMP-2, VEGF	Genepin	[91]
		N. d.	N. d.	Cefquinome sulfate	GA	[123]
		N. d.	N. d.	Tetracycline hydrochloride	GA	[124]
		N. d.	N. d.	Amoxicillin	GA	[125]
		N. d.	N. d.	Phyllanthus urinaria extract	–	[101]
	Microparticles/nanoparticles	Type B	Bovine skin	Bovine serum albumin	–	[126]
				BMP-4, bFGF	Heat	[98]
				Methotrexate	GA	[127]
		Type A	Porcine skin	Tramadol hydrochloride	GA	[128]
		Type A and B	N. d.	BMP-2	GA	[33]
Spray-dry	Microcapsules	Type A	N. d.	Revaprazan	–	[129]
			N. d.	Curcumin	DCMC	[92]
			N. d.	Nifedipine	–	[130]
			N. d.	Valsaran	–	[131]
			N. d.	Fenofibrate	–	[132]
			N. d.	Ibuprofen	–	[133]
			N. d.	Ibuprofen	GA	[134]
			N. d.	Piroxicam	–	[135]
Electrospray	Nanocapsules	N. d.	Tilapia fish skin	Moringa oleifera	–	[136]
	Microparticles/nanoparticles	N. d.	N. d.	Piroxicam	–	[87]
		N. d.	Tilapia fish skin	Bitter gourd	–	[137]
		Type A	Porcine skin	Epigallocatechin 3-gallate	GA	[89]
	Microspheres	Type B	Bovine skin	Human bone marrow stromal cell	CaCl_2_	[96]
			N. d.	Human-adipose-derived stem cells	–	[104]

### 4.1. Desolvation

**Figure 5:**
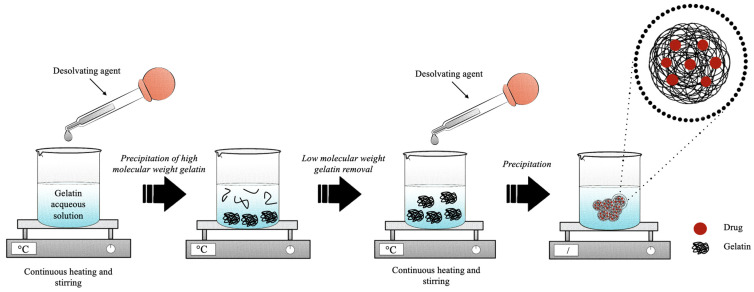
Schematic representation of the production process of gelatin nanoparticles by two-step desolvation method.

The desolvation technique was firstly described in 1978 by Marty et al. for the development of nanoparticles. It consisted of a multistep process, as represented in Figure 5. Generally, in the first step of desolvation, an amount of polymer is dissolved in purified water (0.8–9.0% [81,111]) under continuous heat (35.0–50.0 °C [84,100]). Subsequently, the desolvating agent, usually represented by acetone [77], is added dropwise to induce the sedimentation of the high-molecular-weight (HMW) fractions (1:1 ratio). Low-molecular-weight (LMW) and HMW gelatin are separated because a mixture of the two fractions of different molecular weights affects the dimensional distribution of the final product due to the different gelling properties [111]. In this way, a supernatant is obtained consisting of LMW gelatin and a pellet formed by the HMW polymer. In the second step of desolvation, the supernatant is eliminated, and the resulting pellet is dissolved again in water under continuous heat. Then, the repeated addition of non-solvent, usually in a volume three-times greater than the volume used for the redispersion of high-molecular-weight gelatin, leads to the formation of nanoparticles [109]. Usually, the ratio between solvent and non-solvent in the first desolvation step is 1:1 [106,107], while in the second desolvation step, the amount of non-solvent increases up to three times compared to the solvent [110,111]. Subsequently, to stabilize the precipitated gelatin nanoparticles, the main crosslinker used is 25% (*v*/*v*) GA [79] or, to avoid its toxic effects, 1 M CaCl_2_ is used [105].

However, to have stable and disaggregated gelatin nanoparticles, Coester et al. developed a simple synthesis method based on two-phase desolvation by modifying the classical desolvation process of Marty et al. [108]. Several modifications of the method of Coester et al. have been reported to optimize the process of forming nano-sized gelatin particles (Table 2). The key parameters were found to be temperature, pH, solvent/non-solvent ratio, and the use of crosslinkers.

Temperature is a processing parameter that strongly affects nanoparticles’ synthesis. This is due to the polymer melting temperature, which plays an important role in the formation of the nanoparticles since the increment of the temperature is directly proportional to the size of the final product [105]. The results obtained by Narayanan et al. suggested that 40 °C was the temperature that allowed achieving gelatin particles with an optimal nanometric distribution since temperatures below 35 °C did not allow the formation of particles and temperatures above 40 °C brought the development of large aggregates [105]. This phenomenon could be explained by the intrinsic gelling properties of gelatin for which viscosity is inversely proportional to temperature [77,105]. Due to this, the desolvation process is usually performed at 40 °C [76,77,78,81,86,105,106,107,109,110,111] or 50 °C [79,100] and rarely at 35–37 °C [84].

Besides temperature, also pH plays a fundamental role in gelatin nanoparticles’ synthesis process. Indeed, to maximize the strength of electrostatic interaction, the pH of the formulation in the second desolvation step can be corrected according to the type of gelatin used. Nahar et al. showed that the optimum pH for Type A gelatin was about 3.0, while for Type B gelatin, it was about 11.0 [78]. Azarmi et al. showed that, at a pH value of 4.0 or higher, there was an early agglomeration of the polymer with the addition of the desolvating agent [76]. Their study explained that, at a strongly acidic or basic pH, the polymeric chains are highly positively or negatively charged; therefore, the electrostatic repulsion prevents the uncontrolled agglomeration of the polymeric chains [76].

Nanoparticle size was found to be affected both by polymer concentration and type. Indeed, Nejat et al. demonstrated that the average nanoparticle diameter increased with the polymer concentration and that nanoparticles prepared from Type B gelatin were bigger than those produced from Type A gelatin [77]. These results were also confirmed by Nahar et al. already in 2008 [78], but were in contrast with the results obtained by Azarmi et al. and Wang et al. in the studies conducted in 2006 and 2013, respectively [76,100]. Wang et al. explained that the difference in the obtained diameter of the nanoparticles could be attributed to the higher Bloom number and corresponding molecular weight of Gelatin A compared to Gelatin B [100].

Crosslinkers are used to stabilize the system and to tune the degradation rate of the nanoparticles and the encapsulated drug release kinetics. Generally, GA is used with a concentration between 8% and 25% [81,106]. Only one study reported the use of CaCl_2_ as a crosslinker, not only to help the formation of the nanoparticles in solution, by the electrostatic interaction between the positively charged Ca and the negatively charged deprotonated carboxyl groups at pH 8.5, but also to evade the toxic effect of GA [106]. As regards the ratio between gelatin and crosslinker, it has been reported to influence the nanoparticles’ size. Wang et al. demonstrated an inverse relationship between the amount of crosslinker and the dimension of the nanoparticles, probably because a higher crosslinking degree induces a greater shrinkage of the polymeric network and less swelling [100]. Furthermore, the increase in crosslink density also affected the net surface charge of the nanoparticles, as excessive use of positively charged amino groups increases the overall net negative charge due to the loss of positive charge [100].

### 4.2. Nanoprecipitation

**Figure 6:**
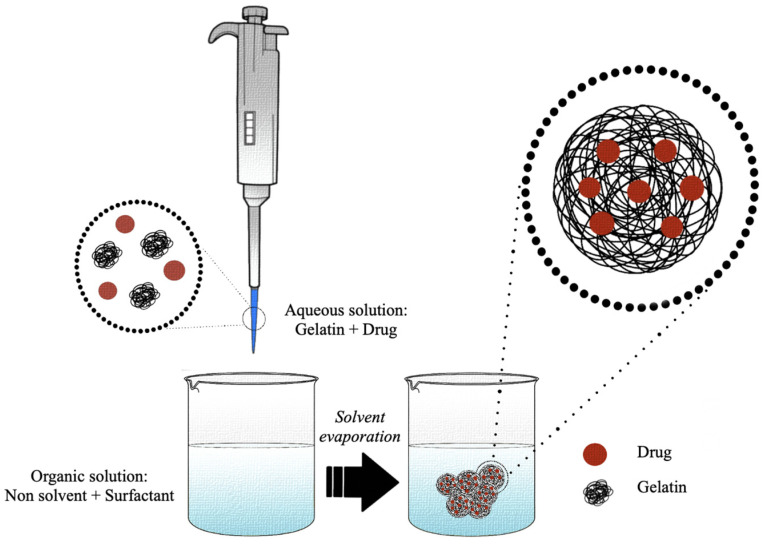
Schematic representation of the production of gelatin nanoparticles by nanoprecipitation method.

The nanoprecipitation technique, which allows obtaining dispersible colloidal particles with a sub-microscopic diameter, was patented by Fessi et al. in 1992 [138]. The generation in solution of the nanoparticles, by means of this method, is explained by the transient interfacial turbulence due to the diffusion of the organic solvent in the aqueous phase [139]. The conditions necessary for the formation of nanometric particles include the reciprocal and total miscibility between the solvents of the two phases so that the resulting mixture consists of a solvent in which the polymer is soluble and a solvent in which the polymer is insoluble [139]. In brief, an organic phase (solvent phase) containing the polymer and the optional surfactant is dropwise mixed, under constant stirring, with an aqueous phase (non-solvent phase) (Figure 6). The protocol can be reversed without altering the formation of the nanoparticles [140].

Compared to other techniques, nanoprecipitation is considered a rapid and straightforward method to obtain stable and monodisperse gelatin nanoparticles [58]. Several protocols based on the nanoprecipitation technique have been pointed out to generate nanometric particles of gelatin for the delivery of several bioactive molecules (Table 3). Generally, a small amount of gelatin (0.8–4.0%, *w*/*v*) is dissolved in deionized or ultra-pure water to generate a solvent phase [102,112]. Then, the gelatin aqueous solution is added dropwise, under mechanical stirring, to an organic phase, usually represented by ethanol, methanol, or acetone [80,114].

Polymer concentration is a key factor. As demonstrated by Khan et al., the increase of the gelatin concentration is directly proportional to the size and polydispersion of the final product [114]. Indeed, the increase of the polymeric solution viscosity is responsible for the solvent phase’s delayed dissolution toward the non-solvent phase [102]. Khan et al. studied how the stability of the nanosuspension varied as the ratio of solvent to non-solvent varied, and they came to the conclusion that a 10- to 20-fold increase in the organic phase compared to the solvent phase allowed a low polydispersion of the final nanoparticles; on the contrary, a decrease of the organic phase compromised its stability [114]. Generally, the ratio of the solvent is kept equal to or greater than 1:10 during the synthesis process [80,82,114], although there are a few exceptions, such as the study conducted by Khan et al. in which it was possible to obtain a monodisperse nanosystem creating a mixture between two organic solvents [112].

To avoid instabilities due to particle aggregation, different types of stabilizers could be added to the non-solvent phase, such as Poloxamer 188, Poloxamer 407 (or Pluronic F-127), Lutrol F-127, Tween 80, or Lutrol F68 [69,80,90,102,113]. However, also the concentration of the stabilizer can influence the dispersion and the hydrodynamic diameter of the gelatin nanoparticles, since it was found that it influences the entry of the polymer into the non-solvent phase. Indeed, Quironz-Reyes et al. demonstrated the existence of a positive correlation between surfactant concentration and nanoparticles’ monodispersion, indicating that an increase of the surfactant concentration is able to decrease the surface tension and viscosity, allowing for stabilization and lower hydrodynamic diameters [102].

The precipitated nanoparticles can be stabilized or not [112] with the use of a chemical stabilizer. Among existing crosslinking agents, also in this case, the most-commonly used crosslinker is GA, with a final concentration of 2–5% (*w*/*v*) [69,113]. The concentration of the crosslinker was found to influence the dispersion and the hydrodynamic diameter of the gelatin nanoparticles. Fathollahipour et al. found that increasing the crosslinker concentration decreases the particle aggregation [113].

### 4.3. Coacervation

The phenomenon of coacervation was first described in the literature in 1929 by De Jong [141]. Coacervation is a term used to describe the formation of colloid-rich liquids resulting from various processes that cause phase separation in aqueous systems of macromolecules or colloids in solution [142]. Colloidal systems are defined as a biphasic system, in which one phase is a continuous liquid, while the other phase is a solid highly dispersed in the liquid in the form of particles or structures derived from them, even smaller than a nanometer [143]. Based on the number of polymers used, the coacervation process in aqueous solution is divided into simple and complex coacervation. Simple coacervation involves a single polymer, while complex coacervation is based on the use of two polymers with opposite charges [144].

Generally, in the simple coacervation process, a prefixed quantity of gelatin is dissolved in water to obtain a polymeric aqueous solution at a fixed concentration (1–10%, *w*/*v*) [94,116]. Subsequently, a surfactant is added to the resulting solution by magnetic stirring or sonication. The coacervating agent, represented by ethanol or propanol [94,116], is added gradually to the aqueous phase, while the addition of the crosslinker, such as 5% (*w*/*v*) GA [116] or 30% (*w*/*v*) formalin [94], occurs following the formation of gelatin microcapsules or nanocapsules.

The concentration of the polymer affects the size and shape of the final product. As reported by Wang et al., there is a direct relationship between the concentration of the aqueous solution and the average distribution of the gelatin capsules. A polymeric concentration lower than 1% (*w*/*v*) leads to a low yield of the final product, while a polymeric concentration higher than 4% (*w*/*v*) induced particle agglomeration, probably because of the increase of the aqueous phase viscosity, which experiences a greater resistance to breaking and deformation compared to a lower-viscosity liquid [116].

**Figure 7:**
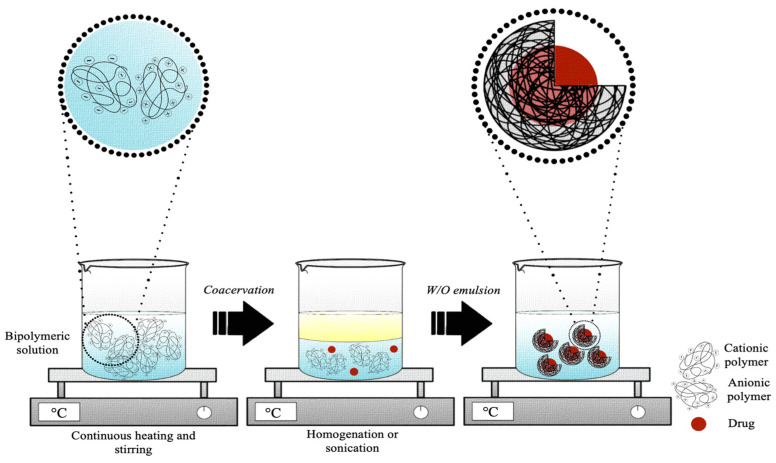
Schematic representation of the synthesis process of gelatin microcapsules or nanocapsules by complex coacervation method.

Although simple coacervation appears to be simpler, in practice, complex coacervation (Figure 7) is mainly used to generate gelatin-based micro- or nano-delivery systems. Variations of the main variable parameters were summarized in Table 4. In summary, the first step includes the formation of a coacervate obtained by the hydration of two or more polymers (0.9–30.0%, *w*/*v*), in deionized or ultrapure water, under continuous stirring and heating to obtain their complete dissolution [83,120]. In the second step, an oily phase is then added to the coacervate obtained to create a water-in-oil emulsion. The oil phase can often be represented by an oil, such as olive oil [118], sunflower seed oil [117], or canola oil [99], or by the bioactive molecule to be encapsulated if it is an oil [85]. Hence, homogenization or sonication is used to achieve a homogeneous emulsion [103,118]. This phase usually contains one or more emulsifiers capable of stabilizing the emulsion. Generally, the mainly used ones are Span 80, Tween 80, and hydroxyethyl cellulose (HEC) [103,117,118]. When the coacervate is formed after the addition of the bioactive ingredient, the coacervation process consists of three phases: first, the hydration in ultrapure or demineralized water of one of the two polymers selected for coacervation, followed by the addition of an oil phase to form a water-in-oil emulsion, and finally, the addition of the second external polymer solution to induce the formation of the coacervate. Once the time of the formation of the microcapsules or nanocapsules has elapsed, the crosslinking occurs in a chemical or enzymatic way, with 0.5–25.0% GA, 20–30 U/g TG, 1.0% CaCl_2,_ or 25.0% FA [85,117,119,120].

Even in complex coacervation, the concentration of polymers plays a decisive role in the size of the final product. As observed by Xing et al., the linear relationship between the two parameters induces a shift in the dimensional order of the particles, passing from nanometric to micrometric dimensions when the concentration of gelatin exceeds 1% [103].

Since the coacervation complex is an electrostatic reaction that occurs between macromolecules with opposite charges, another critical parameter is the pH due to its effect on the overall charge of the polymer. Indeed, the study by Zhang et al. confirmed that, as the pH of the polymer solution increases, a change in the surface charge from positive to negative occurs in conjunction with the isoelectric point [117]. The trade-off for obtaining particles with a small hydrodynamic diameter and better morphology is to improve the strength of the bridge between the polyanionic and polycationic complexes [83]. Santos et al. showed that positive and negative charges’ imbalance in solution affects the final product [99].

### 4.4. Emulsion

**Figure 8:**
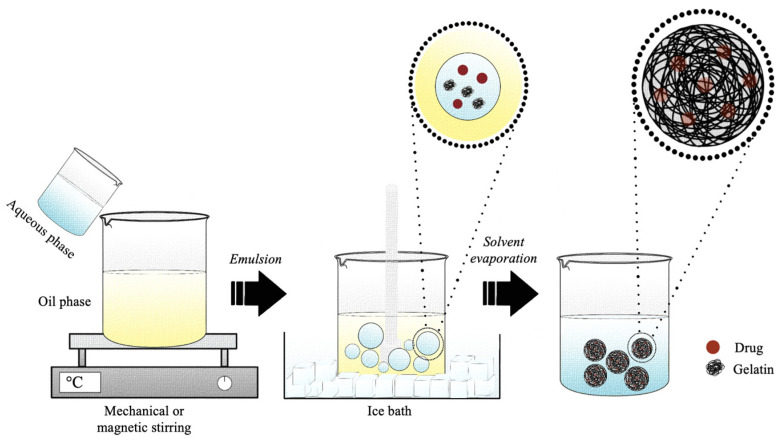
Schematic representation of the production of gelatin microspheres or nanospheres by water-in-oil emulsion method.

The emulsification method is a technique based on the drop-by-drop addition and mixing of an aqueous phase containing the polymer in a non-polar organic (Figure 8) [145]. Standard emulsification methods are based on the preparation of a single water-in-oil (w/o) or a double-water-in-oil-in-water (w/o/w) emulsion and are well-suited to encapsulate different types of lipophilic and hydrophilic drugs [146,147]. Thus, the w/o method is suitable for the encapsulation of hydrophobic compounds (lipophilic agent added to the oil phase), while the w/o/w method is used to encapsulate more hydrophilic molecules (hydrophilic agent to the first water phase). The single- and double-emulsion techniques can be simply scaled up by adjusting the amount of polymer, the type of solvents, the addition of drugs, the type of surfactant, and sonication or homogenization [147]. Emulsions allow developing both micro- and nano-DDSs, but they are often used to develop microparticles in the range 10 to 100 μm [148].

Several protocols based on the w/o emulsion method have been developed and optimized to obtain micrometric or nanometric spheres for the release of several types of bioactive compounds (Table 5). Generally, 1–40% (*w*/*v*) of gelatin is dissolved in demineralized or ultra-pure water to obtain the water phase [122,127]. Then, it is drop-by-drop added, under continuous mechanical or magnetic stirring, to the oil phase, to obtain the formation of the gelatin microcarriers or nanocarriers, containing a single or blends of surfactants, such as sorbitan sesquioleate (SO-15), peg-60 hydrogenated castor oil (HCO-60), Tween 80, Span 80, or Polysorbate 20 [98,101,123,128]. The oil phase is usually represented by olive oil, sesame oil, corn oil, polydimethylsiloxane (PDMS) oil, soybean oil, paraffin liquid, ethyl acetate, and also by polymethyl methacrylate (PMMA) dissolved in a mixture of equal quantities of chloroform and toluene [27,91,93,121,123,126,127,128].

The modifications and optimizations of the emulsion method proposed by Yoshika in 1981 for the formation of microspheric and nanospheric gelatin vectors [121] regarded also the modulation of the working temperature and the use of crosslinkers. Indeed, the homogenization of two phases was performed by preheating both the oil and water phases (3–80 °C) and then cooling down the emulsion in an ice bath (4–5 °C), in order to reverse the aqueous state of gelatin to its gelatinous semi-solid state [124] or by performing the entire homogenization process at 4 °C [127].

Usually, gelatin particle crosslinking can be carried out after the formation of the aqueous phase [93] or at the end of the process, when the final product is obtained [33]. The most-common chemical crosslinking agents are 0.06–50.00% (*v*/*v*) GA, 10% (*v*/*v*) FA, 1% (*w*/*v*) genipin, and MBA [91,93,121,124,127]. Heat can also be used to crosslink gelatin particles, as demonstrated by Nguyen et al. with methacrylated gelatin [98]. The concentration of the crosslinker in turn influences the shape and size of the final product. In particular, it was demonstrated that the concentration of the crosslinker used in the synthesis process is inversely proportional to the average size of the particles; indeed, by increasing the concentration of the crosslinker, particles appeared denser and with a smaller size [127]. In a study carried out by Turner et al., it was shown that the type of gelatin used in the synthesis process strongly influences the properties of the produced particles. Indeed, it was possible to obtain a lower polydispersion and a smaller diameter by using Type A gelatin, thanks to the lower molecular weight and viscosity in solution of the polymer [91].

The surfactant concentration can influence the final product morphology. As demonstrated by Houshyari et al., there was a direct relation between the surfactant concentration and the average size of the particles [128]. In some cases, the surfactant cannot be used to stabilize micro- or nano-gelatin-based systems because it does not allow entrapment of the bioactive molecule to be conveyed. To overcome this problem, Tabata et al. showed how it is possible to obtain a stabilized system without particle aggregation even in the absence of surfactants, by exploiting the gelatin gelling properties at a low temperature and by using an oil organic phase at high viscosity [122].

### 4.5. Spray Drying

**Figure 9:**
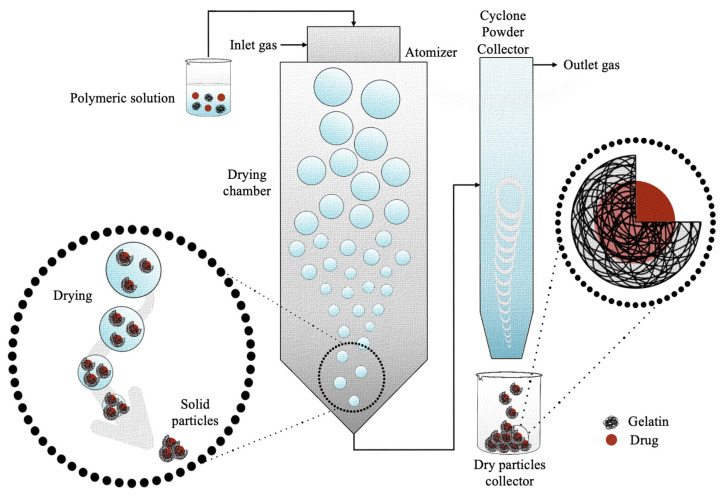
Schematic representation of the production of gelatin microcapsules or nanocapsules by spray drying method.

The spray drying method was first developed and patented by Percy S. in 1872 as a method able to reach a state of “minute division” starting from a substance in liquid or solid form [149]. Thus, spray drying is a dispersion technology able to convert materials from a liquid state to a dry granular state, micro- and nano-sized, by spraying a solution or a suspension in a hot-air-drying system [150]. Since its invention, the spray drying method has been improved over the decades since early devices lacked efficiency and safety. After overcoming these problems, spray drying started to be used firstly for food-industry-related applications and later for the pharmaceutical, chemical, ceramics, and polymer industries [151]. The spray drying process firstly involves the dissolution of the polymer, or two polymers, within known volumes of water to form an aqueous solution. The processing parameters are customized and optimized according to the application and to the polymer/drug properties (Table 6). As regards the production of gelatin nanoparticles, a very wide concentration is used, which was reported to be from 0.004% to 13.330% (*w*/*v*) [92,135]. Since the polymer has a poor solubility in some organic compounds in which the molecules to be transported are soluble, water- and alcohol-based mixtures, such as ethanol or methanol, are often prepared [129,130]. A surfactant such as Tween 80, sodium lauryl sulfate (SLS), or hydroxypropyl methylcellulose (HPMC) is added to lower the surface tension at the interface between the two liquids [92,131,135]. The mixture is stirred magnetically. The resulting solution is then subjected to the spray drying process (Figure 9), which consists of subjecting the liquid suspension to a high pressure and forcing its exit through a nozzle. As a result of the pressure, the liquid suspension comes out in the form of small droplets (atomization), which increase the heat exchange due to the increase in the specific surface. Then, the droplets meet the hot air flux, which makes the liquid evaporate very quickly from the polymer, which is the reason why the solid contained in each drop forms particles, generally hollow inside due to rapid drying. Usually, an inlet and outlet temperature of between 100 and 140 °C [92,129] and 65 and 80 °C [129,132], respectively, a flow rate of 3–10 mL/min [92,131], and an atomizing air pressure of 4–5 kg/cm^2^ [134,135] are used. Generally, the diameter of the nozzle is 0.7 mm.

Similar to the other synthesis techniques, also in this case, the gelatin concentration, surfactant concentration, and crosslinking agent are key processing parameters that strongly influence the particles’ properties. Amon them, the gelatin concentration is the parameter that mostly affects the particle size. As demonstrated by Kocer et al., the concentration of the polymer has a direct relationship with the hydrodynamic diameter of the particles [92]. Indeed, they found that the four-fold decrease of the gelatin concentration causes a change in the average diameter of the obtained microspheres from 3.4 ± 0.4 μm to 1.90 ± 0.09 μm, with a strong reduction of the particle size standard deviation [92]. Moreover, Yousaf et al. demonstrated that the concentration of gelatin can also influence the solubility of the bioactive compound, since a high gelatin concentration worsens the drug solubility when compared to formulations with a lower gelatin content [133]. As regards the surfactant, its presence and amount influences the particles’ development. In particular, the absence of the surfactant was reported to be responsible for the difficulties in handling the particles because of the strong electrostatic interaction, which caused the microparticles’ dry powders to go in all directions, while its presence at low percentages helped the formation of gelatin microspheres and, at higher percentages, hindered their formation [133].

When using the atomization method to obtain gelatin microcapsules, chemical, physical, and enzymatic crosslinking are not always performed to stabilize the final product. However, GA or DCMC have been used. In particular, Yong et al. crosslinked gelatin microcapsules with 25% GA, managing to significantly delay the active principle release [134]. Similarly, Kocer et al. chemically crosslinked gelatin microcapsules with 45 × 10^−5^% (*w*/*v*) DCMC [92].

### 4.6. Electrospray

**Figure 10:**
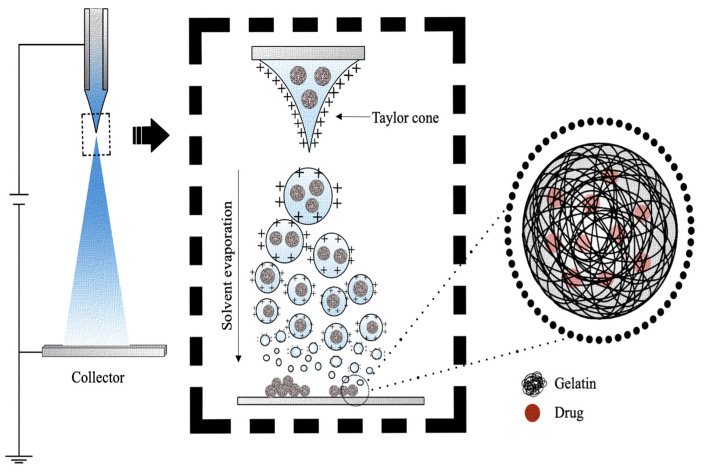
Schematic representation of the production of gelatin microspheres or nanospheres by electrospray method.

Electrospray is a commonly used technique for the development of both microparticles and nanoparticles, where typically, a liquid with non-zero electrical conductivity is expelled from a capillary nozzle, to which a potential difference is applied (Figure 10) [152]. As the solution is ejected through the nozzle, the effect of a high electric field is to generate a mist of highly charged droplets, which deform into a conical shape, commonly known as a Taylor cone [153]. Due to the evaporation of solvents during the course of jet spraying, the particle size decreases as the Rayleigh limit is reached [154]. As described in Rayleigh’s theory of liquid dispersion, when the electrostatic force within a drop exceeds the surface tension at the surface of the drop, it causes the Coulomb fission of the liquid droplets into smaller droplets, which are collected on a collector with an opposite charge to that present on the surfaces of the drop [154,155]. The preparation of polymeric DDSs by electrospray can overcome the limitations of emulsion-based methods such as the use of organic solvents, which can induce the denaturation of protein-based drugs during processing or reduce particles’ polydispersion, which makes the emulsion processes non-reproducible and, thus, difficult to translate to clinical use [156].

B. Vonnegut and R.L. Neubauer were the first to use tension to produce micrometer-sized spherical particles to be sprayed [157]. There are two variants of the electrospray technique, which differ in the way the drops are collected, which are plate electrospray and in-solution electrospray. Plate electrospray involves the collection of individual or groups of charged droplets in a grounded plate. Alternatively, in-solution electrospray involves the collection of charged droplets in a beaker containing the crosslinking solution [158].

The electrospray preparation protocol is optimized and customized according to the gelatin and solvent type (Table 7). Typically, a predetermined amount of gelatin (0.5–8.5%, *w*/*v*) is dissolved in distilled water, 0.01 M phosphate-buffered saline (PBS), or 20–30% (*v*/*v*) acetic acid under continuous magnetic stirring [89,96,104,137]. The resulting gelatin solution is sprayed (Figure 10) under high voltage (6–21 kV) and at a constant flow rate of 0.12–20.00 mL/h [104,136]. Generally, the distance between the nozzle and the target is from 3 cm to 10 cm [89,97]. As regards chemical crosslinkers, usually 5 % (*w*/*v*) GA [89] or 2–3% (*w*/*v*) CaCl_2_ is used [89,104]. The concentration of the crosslinker was found to influence the quantity of the obtainable microspheres. A study by Xu et al. demonstrated how a concentration of CaCl_2_ equal to 3% (*w*/*v*) was able to produce a greater number of microspheres than 1 % (*w*/*v*) CaCl_2_ [96].

The size of the microspheres can be predicted by the Hartman scaling law and can be controlled by adjusting the applied voltage and flow rate [104]. The size of the sphere is directly proportional to the flow rate of the liquid and the concentration of the polymer solution, while it is indirectly proportional to the voltage and electrical conductivity of the solution [104]. As confirmed by the studies of Hani et al., an excessively high concentration of gelatin unbalanced the viscoelastic forces and the surface tension repulsion, resulting in the formation of mixed spherical fibrous structures, rather than nanospheres [136]. On the contrary, as the concentration decreases, the repulsion of the surface tension prevails over the viscoelastic forces, forming uniform particles of a spherical shape [136].

## 5. Preclinical and Clinical Outcomes

### 5.1. Preclinical Studies

Many gelatin-based micro- and nano-sized DDSs, developed through the aforementioned synthesis techniques for many applications, have shown promising results in vitro and have undergone preclinical trials. As listed in—but not limited to—Table 8, gelatin-based DDSs were found to be preclinically evaluated for the treatment of several kinds of diseases belonging mainly to the digestive (e.g., liver cirrhosis [159], tumor [160,161], peritoneal tumor [162], peritoneal fibrosis [163], pancreatic tumor [164,165]), visual (e.g., infections [106], glaucoma [107], vitreoretinopathy [166], corneal neovascularization [167]) musculoskeletal (e.g., osteonecrosis [168], osteoarthritis [169,170]), urogenital (e.g., bladder [171] and ovarian [172] cancer), respiratory (e.g., lung cancer [173,174]), immune (e.g., inflammation [105,175]), and peripheral nervous (e.g., resected nerve [176]) systems.

As regards the urogenital apparatus, the efficacy of drug-loaded gelatin microparticles was evaluated against bladder [171] and ovarian cancer [172]. Indeed, the study conducted by Lu et al. [171] demonstrated that, to overcome the limitation of drug penetration into the bladder tissues, the use of gelatin-based nanoparticles loaded with chemotherapeutic agents such as paclitaxel brought many advantages in intravesical bladder cancer therapy, since they exhibited a significant anticancer activity against bladder cancer cells, besides a higher drug concentration in the tissue than the commercial preparation (pacliaxel/Cremophor/EtOH formulation) [171].

Similarly, DeClercq et al. demonstrated how the prolonged permanence of paclitaxel in the abdominal cavity of mice through its slow release from gelatin-based microparticles improved the survival rate with a significant reduction of peritoneal carcinomatosis recurrence in ovarian cancer [172]. Drug-loaded gelatin microparticles’ efficacy was evaluated also against liver cancer [161]. In particular, cisplatin-loaded gelatin-based microparticulate formulations not only showed no apparent adverse systemic effects (probably due to the 37-fold reduction in the cisplatin dose compared to the commonly used free dose of cisplatin), but also a much more pronounced antitumor effect in rabbit with hepatocellular carcinoma [161]. Furthermore, it was also hypothesized that probably the degradation of the polymer causes a reduction in particle size, leading to slow capillary motility and a consequent increase of cisplatin in the tumor, leading to a longer-lasting effect [161]. Similar results were reported also by Nitta et al. and Gunjii et al. using cisplatin-loaded gelatin microspheres for liver tumor and peritoneal carcinomatosis, respectively [160,162]. Indeed, the first study showed that in rabbits treated with cisplatin-containing gelatin microspheres in combination with flavopiridol, the tumor proliferation rate was 54% compared to the control group, which was close to 600% [160]. However, the second study demonstrated that the slow and controlled release of cisplatin encapsulated in gelatin microspheres into the tumor site significantly potentiated the drug antitumor effect [162]. An increase in the survival time of mice treated with the cisplatin-loaded gelatin microspheres of about 25 days compared to mice treated only with free cisplatin was registered, revealing DDSs’ ability to significantly reduce the free drug systemic adverse effects (hematotoxicity and nephrotoxicity) [162]. To increase specific drug delivery and reduce possible side effects caused by a non-specific distribution, Ching-Li Tseng et al. [173] generated a site-specific recognition system by surface functionalization of gelatin nanoparticles, initially loaded with cisplatin, with biotinylated epidermal growth factor (bEGF), thus increasing the antitumor concentration level in lung cancer cells with a high bEGF receptor (EFGR) expression compared to those with low EGFR expression. Additionally, the active targeting nanoparticle systems also exhibited lower toxicity, likely due to lower prevalence in the systemic circulation and the tendency to concentrate in cancerous lung tissue [173].

The use of gelatin-based microsystems or nanosystems for cytokine delivery was preclinically investigated for the treatment of proliferative vitreoretinopathy [166], inflammatory disease [175], and bone, nerve, and liver regeneration [159,168,176]. As demonstrated by Hirose et al., the use of such systems for the administration of bioactive proteins, such as bFGF and beta interferon (IFN-β), is a promising strategy for the treatment of proliferative vitreoretinopathy, with significant advantages compared to other models [166]. Gelatin microsystems, in addition to being biodegradable and not causing undesirable effects, also allowed host cells to be stimulated through a reproducible sustained release system [166].

The VEGF-loaded gelatin microspheres in a synthetic scaffold enabled synergistic effects in promoting the attachment, proliferation, and osteogenic and angiogenic activities of bone marrow tissue mesenchymal stem cells by exhibiting greater activity than scaffolds without beads [168]. Furthermore, in a rabbit model of glucocorticoid-induced femoral head osteonecrosis, these scaffolds effectively promoted new bone formation in the damaged implant canal [168,175]. The study demonstrated that, despite the introduction of new effective treatments to prevent the onset of colitis or reduce mucosal damage in experimental models, such as interleukin-10 (IL-10) gene transfer or the administration of engineered bacteria that secrete IL-10, the use of gelatin-based microparticle systems for cytokine delivery, for the treatment of inflammatory bowel disease, was shown to be more suitable for clinical application than other systems because the control and delivery of an optimal dose of IL-10 can be achieved without a significant increase in blood cytokine levels, with significantly reduced side effects [175].

As bFGF plays an important role in the regeneration of peripheral nerve defects by affecting nerve cells, Schwann cells, and fibroblasts and promoting axonal outgrowth from the proximal nerve stump, to eliminate the problem due to its short half-life in vivo, Matsumine et al. preclinically evaluated the efficacy of bFGF-loaded gelatin microsystems for peripheral nerves’ regeneration, confirming how the use of such DDSs leads to a significant increase of the nerve regeneration rate, in terms of the number and degree of maturation of nerve axons [176]. Finally, Oe et al. further confirmed the potential of gelatin microspheres as a promising technology to enhance the in vivo therapeutic effects of growth factors. In particular, he demonstrated that the controlled administration of hepatocyte growth factor (HGF) allowed efficient recovery from liver fibrosis in experimental mice by stimulating liver regeneration [159]. The histological and biochemical observations were statistically significant compared to the injection of free HGF or the use of unloaded gelatin microspheres, most likely due to the maintenance of high blood levels of HGF, which improve liver function due to slow polymer degradation [159].

Saravanan et al. investigated the delivery of anti-inflammatory pharmaceutical agents using magnetic gelatin microparticles [169]. In preclinical research, controlled release systems of diclofenac sodium were developed in which the target site can be controlled exogenously by introducing magnetite into gelatin microspheres. This not only allowed for the specific targeting of the drug, but also for the improvement of the release rate thanks to an applicable external stimulus [169]. The use of moxifloxacin encapsulated in gelatin nanoparticles developed by Mahor et al. showed significant effects in reducing discharge, redness, swelling, and infection compared to the marketed MoxiGram^®^ for the treatment of bacterial eye infection [106]. Comparing the two formulations, the experimental analysis performed on rabbits revealed significant antibacterial activity when the nanosuspension was used compared to the commercially available formulation. Indeed, while with the latter, despite the high doses of administration, there was no significant effect six days after the treatment, with the controlled release system, the relief of symptoms was obtained in four days with a regimen of administration of twice a day [106].

Shorky et al. compared the pharmacological effects of timolol maleate encapsulated in gelatin nanoparticles with commercially available formulations for the treatment of glaucoma [107]. The reported data showed that, although this drug had a poor corneal penetration and induced eye irritation, its encapsulation in gelatin-based nano-formulations made it an excellent candidate for the treatment of glaucoma. In particular, the preclinical study demonstrated that the pharmacological effects of loaded nanoparticles were superior to those of commercially available timolol maleate eye drops due to a 10-fold loss of the original concentration after only 20 min of administration because of tearing [107]. On the contrary, thanks to the multiple properties of the polymer, including the properties of adhesion to the mucosa, which facilitated the interaction with the intraocular cavities, the residence time of the active agent was found to be longer when the timolol maleate was encapsulated in the gelatin nanoparticles. Advantageous results of a gelatin nano-sized formulation for ophthalmic use were also obtained by Yu-Lun Chuang et al. in 2019 [167]. The study enabled the generation of a nano-sized controlled release system of kaempferol (KA) using gelatin as a polymeric material. Again, the nano-formulation increased the bioavailability of the drug compared to the preclinical tests conducted with pure KA, demonstrating that the nanosuspension allowed having a significant therapeutic effect against the corneal neovascularization process, besides having an anti-angiogenic effect [167].

To overcome the disadvantage of the short plasma half-life of ibuprofen sodium and repeated oral or intravenous administration to maintain a therapeutic dose in vivo for the treatment of acute inflammation, Narayanan et al. studied the behavior of the anti-inflammatory drug encapsulated in gelatin nanoparticles [105]. The preclinical study showed that the nano-formulation did not cause significant side effects (i.e., no cytotoxicity, immunocytotoxicity) and that the performed surface modification with polyethylene glycol (PEG) created a barrier that reduced drug adsorption while providing a longer circulation time [105]. Indeed, by comparing the plasma concentrations of the encapsulated and free drug, it was observed that the nano-DDS provided a significant sustained release up to 96 h, contributing to the slow and continuous release of the bioactive compound with enhanced pharmacokinetics, thus increasing the duration and efficacy of the treatment [105]. Similarly, the pharmacokinetic and pharmacodynamic studies conducted by Kumar et al. showed the significant anti-inflammatory activity of indomethacin-loaded gelatin nanoparticles for the treatment of acute inflammation, with 500% increased bioavailability compared to the free pure drug [170].

The controlled release technology of plasmid DNA or oligonucleotides from gelatin-based DDSs is a promising strategy for tumor suppression. As demonstrated by Kushibiki et al., gelatin microspheres incorporating NK4 plasmid introduced into the subcutaneous tissue of mice inoculated with pancreatic tumor cells extended their survival by 30 days [165]. In contrast, treatment with empty beads, saline, or free plasmid DNA resulted in 50-day survival. The preclinical study found that the NK4 plasmid DNA was released within 28 days at the target site after polymer degradation, showing a good temporal condition between plasmid release and gene expression, while injected free NK4 plasmid DNA was excreted faster [165]. Researchers demonstrated that the expression time of plasmids can be extended by modulating their release time. Indeed, a controlled release of them would prevent DNA degradation by protecting it from DNase attacks and, thus, facilitate cellular transfection [165]. However, changing the route of administration of the plasmid-DNA-loaded gelatin microspheres from subcutaneous to intraperitoneal resulted in no significant differences in the survival rates of the mice compared to the saline- or free-vector-treated group, possibly due to the presence of immunocompetent cells, which in turn caused faster polymer degradation [165]. The introduction of PEG molecules on the surface of the particles increased the efficiency of the system. As demonstrated by Kaul et al., plasmid DNA encoding for b-galactosidase (pCMV-b)-loaded pegylated gelatin nanoparticles showed higher transfection efficiency than the same non-pegylated system, thus increasing gene expression in tumor tissue, attributing these excellent results to the efficient transport system in terms of biocompatibility, biodegradability, and long bioavailability [174]. Non-viral vectors have several advantages over viral vectors, such as low toxicity and low immune response and a lack of integration into the genome [177] As demonstrated by Obata et al., SiRNA-encapsulated gelatin microspheres constitute a promising new therapeutic system for the continuous and controlled delivery of non-viral vectors [163]. Since the siRNA is negatively charged and the gelatin particles are positively charged, there is an electrostatic interaction that protects the siRNA from nuclease degradation. The preclinical study in rats demonstrated the ability to suppress the progression of peritoneal fibrosis using a polymeric microsystem [163].

### 5.2. Clinical Studies

Although gelatin-based DDSs showed promising in vitro and in vivo results, their clinical evaluation is still in the early stages. To date, clinical trials performed using gelatin-based DDSs have been performed with both empty and drug-loaded gelatin microspheres.

As listed in Table 9, empty gelatin microspheres were successfully used for the preoperative treatment of embolization [178,179,180,181,182,183,184,185,186,187,188,189,190,191,192,193]. Despite the approval of the FDA, the use of polyvinyl alcohol (PVA) particles in embolotherapy causes adverse effects, such as vascular or catheter occlusion, due to the dimensional variability and surface irregularity of the particles [182]. On the contrary, the use of trisacryl gelatin microspheres as a new embolic material seems to be more effective in the treatment of tumors and arteriovenous malformations [178], in the preoperative embolization of meningiomas [179], bone neoplasms [180], hemorrhoidal disease [181], and uterine artery embolization for symptomatic fibroids [182,183,184,185,186,188,189,190,191,192,193]. The different aggregation ability was attributed to the different biomechanical properties of the two materials in terms of the surface, deformability, and regularity of the shape [179]. The PVA particles appeared to have a wide shape and size distribution, which are responsible for their aggregation increase [181,182]. Conversely, the smooth and hydrophilic surface, the deformability, and the lack of aggregation made the trisacryl gelatin microspheres an ideal embolic agent [181]. Moreover, several studies confirmed trisacryl gelatin microspheres’ greater penetration capacity and easier injection with a lower occlusion rate [178,179,181]. Beaujeux et al. showed that, with the use of trisacryl gelatin microspheres, there was a linear correspondence between the diameter of the particle and the diameter of the obstructed vessels, thus giving an advantage when the main objective is occlusion [178]. The results of the clinical study revealed that the diameter of the microspheres is related to the type of disease and that the tissue effects of embolization are directly related to the particle size, so that the smaller the diameter of the particles, the greater the probability of tumor necrosis. The safety of embolotherapy can be maintained with the use of microspheres even when the procedure is difficult to perform [178]. Studies conducted by Basile et al. and Bendszus et al. showed how trisacryl gelatin microspheres could be useful also in the preoperative embolization of bone neoplasms and meningiomas [179,181] since they demonstrated a slowed tumor revascularization rate [181] and a deeper penetration into the intratumoral vascular bed than PVA particles, thereby reducing significant intraoperative blood loss [179]. A recent prospective randomized phase III study carried out by Küçükay et al. compared the effect of the different sizes of trisacryl gelatin microspheres in the embolization of the superior rectal artery for symptomatic hemorrhoids. The results showed that the use of trisacryl gelatin microspheres was a safe and effective procedure, with no deaths or severe adverse complications, and that the increase in the micrometric size of the particles resulted in better control of bleeding over 12 months of follow-up, with a low incidence of postoperative pain and a low rate of ischemic complications [180]. Several clinical studies have been performed also for the treatment of uterine leiomyomas, demonstrating an advantage over PVA microspheres. As demonstrated in the phase I study by Spies et al., trisacryl gelatin microspheres are an effective and safe embolization agent [182] as the results showed a 92% reduction in pelvic pain and discomfort without severe complications [182]. Furthermore, the clinical study of Pelage et al. reported that all performed procedures were successful and a complete resolution of 85% of the menorrhagia was registered after 24–48 months of follow-up [183]. Hence, micrometric systems based on trisacryl gelatin conferred an advantage for uterine artery embolization for symptomatic fibroids over non-spherical particles of PVA since they were revealed to be able to target the fibroid more specifically and minimize ischemic damage of normal myometrium and ovaries [185]. Among trisacryl microparticles’ applications, the treatment of postoperative pelvic pain was one of the most-difficult problems to manage, and despite the good clinical success rate of them [186] and the lower incidence of post-treatment tumor enlargement [190], there were no objective and subjective differences in post-procedural pain comparing patients treated with trisacryl gelatin microspheres and PVA particles [184]. A recent study by Han et al. showed that, although there was no significant difference between the two embolization agents, non-spherical polyvinyl alcohol particles produced a greater inflammatory response, a greater prevalence of transient global uterine ischemia of the normal myometrium, and a higher rate of the use of emergency analgesics [194].

Conversely, despite several clinical trials not having reported differences in treatment efficacy [189], postprocedural pain, quality of life, tumor infarction, and other secondary endpoints after one-year follow-up using PVA microspheres and trisacryl gelatin microspheres, arguing that PVA microspheres can induce adequate uterine tumor infarction when the appropriate size and endpoint are used [191], trisacryl gelatin microspheres are always preferred as an embolic agent for the treatment of uterine artery embolization for symptomatic fibroids [188].

Besides being successfully used as an embolic agent, gelatin microparticles and nanoparticles demonstrated their potential as controlled release systems of bioactive molecules for the treatment of several kinds of diseases (Table 10).

In particular, cisplatin-loaded gelatin microspheres were successfully proposed as a new embolization and anticancer material [194]. Indeed, in cases of metastatic liver cancer, the cisplatin-loaded gelatin microsystems were revealed to be able to reduce tumor size by 32%, with mild side effects [194]. Using the same system, clinical results obtained by Tomaya et al. demonstrated the tolerability, utility, and safety of cisplatin-loaded gelatin microspheres also in patients with advanced hepatocellular carcinoma, achieving a 100% success rate, low related complications, and no deaths [195]. However, it should be noted that, although the procedure did not cause serious side effects, 26–52% of treated patients developed post-chemoembolization syndrome [195].

The Ruthin-loaded gelatin nanocarriers revealed their potential as multifunctional component for sunscreen and chemopreventive formulations since a clinical study on the effect of Ruthin-loaded gelatin nanocarriers on human skin revealed that the association between the polymer (gelatin) and the antioxidant (Ruthin) was able to increase the free-radical-scavenging rate to 74% compared with free Ruthin, while the addition of chemical filters induced a 48% increase in Sun protection factor [109]. Therefore, the study demonstrated that the encapsulation of Ruthin in polymeric nanoparticles has a distinct, safe, and advantageous behavior.

The bFGF-loaded gelatin microspheres were instead employed for the treatment of peripheral arterial diseases (e.g., critical limb ischemia, intermittent claudication). The sustained release of bFGF from gelatin microspheres was able to increase blood flow in ischemic limbs with complete or partial regression of ischemic ulcers, with no focal inflammation at the injection site and no systemic effects [196,197,198]. However, some side effects were registered by Kumagai et al. attributable to the topical administration of prolonged release of bFGF, such as the increase of creatinine, aspartate aminotransferase, and the alanine aminotransferase level [197].

Kushara et al. performed a randomized study to improve tissue survival in Subzone II fingertip amputations by comparing standard non-topical treatment with the application on the cut surfaces of bFGF-loaded gelatin microspheres [198]. The study demonstrated that the application of the micrometric bFGF-loaded gelatin system did not lead to an increase in tissue survival, but that the sustained release of the growth factor may be beneficial for the survival of marginal tissues, where microvascular repairs are more complex [199].

Gelatin-based particle microsystems, in addition to being used as delivery systems for anticancer agents, antioxidant molecules, and growth factors, as described above, can also be used as microcarriers for cells. Liu et al. developed an innovative and rapid process, named “bioreactor microcarrier cell culture system” (Bio-MCCS), which allowed using porous gelatin microspheres as a transport system for primary cell cultures [200]. The pilot study compared the effects of a single application of autologous keratinocytes grown in the gelatin microsystems with a single transplant of keratinocyte monolayers on collagen pads for the regeneration of chronic venous leg ulcers [200]. The results showed that the use of collagen pads lengthened the healing time, while the use of the gelatin microparticles induced a faster healing during the first two weeks of treatment [200]. However, due to the lower healing rate in the wound area after the two weeks of treatment, repeated applications were performed to speed up the healing time, and it was found that they were able to induce total healing without [200]. In the same year, Liu et al. conducted another clinical study based on the use of autologous-melanocyte-loaded Bio-MCCS for the treatment of vitiligo or piebaldism [201]. The results obtained showed the success of the implant in a patient who achieved skin repigmentation for more than eight months with no side effects, nor infections or scarring [201].

**Table 10:** Summary table of applications and outcomes obtained with the use of loaded gelatin microparticles/nanoparticles in clinical studies.

Issue	Bioactive Compound	Outcome(s)	Ref.
Chronic venous leg ulcers	Keratinocytes	Fast healing and complete regeneration.	[200]
Vitiligo	Melanocytes	Complete repigmentation, no adverse events.	[201]
Sun protection	Ruthin	Increased free-radical-scavenging rate.	[109]
Metastatic liver tumors	Cisplatin	Reduction in tumor size, no serious side effects.	[194]
Hepatocellular carcinoma	Cisplatin	No serious side effects, 100% success rate, reduced abdominal pain.	[195]
Limb ischemia	bFGF	Complete or partial regression of ischemic ulcers, no local and systemic effects.	[196]
Peripheral arterial disease	bFGF	Improvement in symptoms and no serious complications, incomplete necrosis or ulcer healing.	[198]
Fingertip amputation	bFGF	No statistically significative improvements.	[199]
Limb ischemia	bFGF	No serious adverse events.	[197]

## 6. Conclusions and Future Perspectives

Among the natural biopolymers used in the production of DDSs, gelatin is a promising multifunctional candidate due to its innumerable advantageous properties such as biocompatibility, biodegradability, and low immunogenicity. The gelatin-based systems for the delivery of bioactive molecules find application in the biomedical, cosmetic, and pharmaceutical sectors, especially in the field of nanomedicine. This review offered an overview of the main synthesis methods of gelatin microparticles and nanoparticles from a methodological and mechanistic point of view, while discussing the effect of the different parameters of each process, in order to obtain a more monodisperse and stable final product. Considerable attention was paid to the preclinical and clinical application of gelatin-based DDSs. Many preclinical studies have demonstrated that the release of bioactive molecules encapsulated in gelatin microparticles and nanoparticles improves their local bioavailability, showing promising results for the treatment of tumor pathologies and for tissue regeneration. While important clinical goals have been achieved using the microsystems as a primary embolization method, only recently have gelatin microparticles and nanoparticles been proposed as effective vectors for the controlled release of bioactive compounds. Currently, there are commercial suppliers producing effective gelatin microspheres only for the embolization method, but more research is still needed to achieve excellent results for gelatin-based DDSs for future applications. Overall, the continuous experimental research of gelatin-based microparticles and nanoparticles raises the challenge of obtaining an adequate delivery system that allows a controlled, sustained, and specific release to the target site with the consequent scalability of the production process at an industrial level, not only in the biomedical and pharmaceutical fields, but also in the cosmetic and food sectors.

## Figures and Tables

**Table 2:** Main variable parameters for the fabrication of gelatin-based nanoparticles using the two-step desolvation technique.

Polymer % (*w*/*v*)	Temperature (°C)	Solvent:Non-Solvent Ratio	pH	Crosslinker % (*v*/*v*)	Ref.
0.06	40	N. d.	2.5	GA, 25%	[106]
0.8	40	N. d.	4.0	GA, 8%	[81]
2.0	40	1:3	3.0–11.0	GA, 25%	[78]
2.0	40	1:3	<4.8, 9.2–9.4	GA, 25%	[77]
2.0	40	1:3	3.0	GA, 25%	[110]
3.0	40	N. d.	8.5	CaCl_2_, 1 M	[105]
5.0	40	1:1.6	3.0	GA, 25%	[86]
5.0	N. d.	1:1.6	2.5	GA, 8%	[108]
5.0	40	1:3	2.5	GA, 25%	[107]
5.0	50	1:3	2.5	GA, 25%	[100]
5.0	50	1:3	2.5–12.0	GA, 25%	[79]
5.0	35–37	1:1	N. d.	GA, 25%	[84]
5.0	40	1:3	2.5–12.0	GA, 25%	[76]
7.7	40	1:3	11.0	GA, 25%	[109]
9.0	40	1:3	3.0	GA, 25%	[111]

**Table 3:** Main variable parameters for the fabrication of gelatin-based nanoparticles using nanoprecipitation technique.

Polymer % (*w*/*v*)	Solvent:Non-Solvent Ratio	Non-Solvent	Stabilizer % (*w*/*v*)	Crosslinker % (*w*/*v*)	Ref.
0.80–3.41	1:15	Ethanol	0.80–3.41 Tween 80	GA, 5.0%	[102]
0.90	1:1.5	Ethanol	0.03 Lutrol F68	GA, 0.5%	[90]
1.25	1:20	Ethanol	2.00 Pluronic F-127	GA, 5.0%	[113]
1.25	1:20	Ethanol	2.00 Pluronic F-127	GA, 5.0%	[82]
1.25	1:10	Acetone	4.00 Poloxamer 407	GA, 2.0%	[80]
2.00	1:15	Acetone	3.00 Poloxamer 188	Diisopropylcarbodiimide, 1.50%	[95]
2.00	1:15	Acetone	2.8 Poloxamer 188	GA, 2.0%	[69]
2.00	1:15	Ethanol	4.27 Pluronic F-127	GA, 5.0%	[113]
0.2	1:10	Ethanol	7.00 Lutrol F127	GA, 2.0%	[114]
4.00	1:6	Acetone-dimethyl formamide	–	–	[111]

**Table 4:** Main variable parameters for the fabrication of gelatin-based microcapsules or nanocapsules using coacervation technique.

Type	Polymer % (*w*/*v*)	Gelatin:Polymer Ratio	Surfactant	Coacervant Agent	Crosslinker % (*w*/*v*)	Coacervate pH	Ref.
Simple	1.0–2.0	–	Tween 60	Ethanol	GA, 5.0%	–	[116]
	10.0	–	–	Propanol	Formalin, 30.0%	–	[94]
Complex	0.9	1:1	–	Geraniol	GA, 25.0%	4.45	[85]
	30.0	1:1	Span 80	Fish ω_3_ fatty acid	CaCl_2_, 1.0%	7.00	[120]
	7.5	1:1	Span 80	Moxa oil	FA, 25.0%	4.00	[119]
	0.2	1:1	HEC	-	GA, 0.5%	4.20	[103]
	1.0	1:1	Tween 80	-	GA, 25.0%	4.50	[83]
	1.0	9:1	Span 80 Tween 80	Sunflower seed oil	TG, 20 U/g	4.50	[117]
	1.0	6:1	–	Canola oil	TG, 30 U/g	4.00	[99]
	1.0	1:2	Span 80	Olive oil	–	–	[118]

**Table 5:** Main variable parameters for the fabrication of gelatin-based microspheres or nanospheres using water-in-oil emulsion technique.

Polymer %	Oil	Temperature (°C)	Surfactant	Crosslinker %	Ref.
30	Sesame oil	70–80	HCO-60 SO-15	FA, 10.00%	[121]
40	PMMA in Chloroform/toluene	4	–	GA, 25.00–8.00%	[127]
N. d.	Olive oil	40	–	GA, 0.06–0.12%	[125]
N. d.	Olive oil	50–70	Span 80	–	[101]
11.1	Olive oil	N. d.	–	GA, 10–40 mM	[33]
11.1	Olive oil	45	–	Genipin, 2.0%	[27]
10	Olive oil	45	–	GA, 0.002–0.010 M	[122]
10	Soybean oil	60	Span 80	GA, 50.00%	[124]
10	Liquid paraffin	60	Span 80	GA, 1.30%	[125]
10	Corn oil	37, 4	Polysorbate 20	GA, 10 mM	[98]
2.5	Corn oil	40	–	–	[126]
6	PDMS oil	40	–	Genipin, 1.00%	[91]
15	Soybean oil	50	–	MBA, 0.015 M	[93]
1	Ethyl acetate	50	Span 80 Tween 80	GA, 25.00%	[128]
25	Liquid paraffin	50	Span 80	GA, 25.00%	[88]

**Table 6:** Main variable parameters for the fabrication of gelatin-based microcapsule or nanocapsules using spray drying technique.

Solvent	Polymer % (*w*/*v*)	Inlet T (°C)	Outlet T (°C)	Flow Rate (mL/min)	Pressure of Spray Air (kg/cm^2^)	Surfactant % (*w*/*v*)	Crosslinker %	Ref.
Water	0.004	140	N. d.	3	N. d.	Tween 80, 0.05%	DCMC, 4.5 × 10^−4^%	[92]
	2	130	80	10	5	HPMC, 2.00%	–	[131]
Water-Ethanol	4	100–120	N. d.	5	4	SLS, 0.60%	–	[133]
	5.7	105	N. d.	5	4	SLS, 0.60%	GA, 25%	[134]
	5.7	105	N. d.	5	4	SLS, 0.60%	–	[130]
	8	120	65–70	7	4	–	–	[132]
	13.33	105	N. d.	5	5	SLS, 0.60%	–	[135]
Water-Methanol	0.25	100	65	5	4	–	–	[129]

**Table 7:** Main variable parameters for the fabrication of gelatin-based microspheres or nanospheres using electrospray technique.

Solvent	Polymer % (*w*/*v*)	Flow Rate (mL/h)	Voltage (kV)	Distance (cm)	Crosslinker % (*w*/*v*)	Ref.
Acetic acid	8.5	0.5	20	N. d.	–	[137]
	7.5	0.5	20	10	–	[136]
	4	14.4	21	10	–	[89]
	4	0.12	20	10	GA, 5%	
Water	0.9	1	12.5	N. d.	–	[87]
	0.5	N. d.	8	N. d.	CaCl_2_, 3%	[98]
	0.5	20	6.5	3	CaCl_2_, 3%	[97]
	2.0–4.0	20	6.0–9.0	3	–	[104]

**Table 8:** Preclinical studies on gelatin-based delivery systems for musculoskeletal, gastrointestinal, urinary, circulatory apparatus, and others. The DDSs’ specifications (particles size—micro, M, or nano, N—drug type, target tissue, animal model employed) and outcomes are reported.

Body Apparatus	Application	DDS	Drug	Animal Model	Outcome(s)	Ref.
Digestive	Hepatocellular carcinoma	M	Cisplatin	Rabbit	No adverse systemic effects; more pronounced antitumor effect.	[161]
	Liver tumor	M	Cisplatin	Rabbit	Tumor proliferation almost 6-times lower.	[160]
	Peritoneal carcinomatosis	M	Cisplatin	Mouse	Longer survival time.	[162]
	Peritoneal fibrosis	M	Cisplatin	Mouse	Peritoneal fibrosis progression suppression.	[163]
	Liver cirrhosis	M	HGF	Rat	Enhanced tissue regeneration.	[161]
	Pancreatic cancer	N	Gemcitabine	Mouse	Enhanced antitumor efficiency.	[164]
	Pancreatic cancer	M	NK4 plasmid DNA	Mouse	Angiogenic inhibition and tumor suppression, limit of the route of administration.	[165]
Visual	Eye infection	N	Moxifloxacin	Rabbit	Non-irritant to the ocular tissues, safe, antibacterial power more effective than commercial products.	[106]
	Glaucoma	N	Timolol maleate	Rabbit	Enhanced effectiveness compared to commercial products, non-irritating.	[107]
	Proliferative vitreoretinopathy	M	b-FGF/IFNβ	Rabbit	No side effects.	[166]
	Corneal neovascularization	N	Kaempferol	Mouse	Anti-angiogenic effect, enhanced drug bioavailability.	[167]
Musculoskeletal	Osteonecrosis	M	VEGF	Rabbit	Effective promotion of new bone formation.	[168]
	Osteoarthritis	M	Diclofenac	Rabbit	Specific targeting with external stimuli.	[169]
	Osteoarthritis	N	Indomethacin	Rat	Side effects’ reduction, drug bioavailability increases of 500%.	[170]
Urogenital	Bladder cancer	N	Paclitaxel	Dog	Rapid release, significant increase in antitumor activity, higher tissue concentrations than the commercial formulation.	[172]
	Ovarian cancer	M	Paclitaxel	Mouse	Tumor size reduction.	[172]
Respiratory	Lung cancer	N	EGF	Mouse	Increment of site-specific drug concentration, lower toxicity.	[174]
	Lung cancer	N	pCMV-b	Mouse	Transfection efficiency increase.	[174]
Immunitory	Inflammation	N	Ibuprofen Sodium	Rat	No side effects, increase of drug bioavailability.	[105]
	Inflammatory bowel disease	M	Cytokine	Mouse	Side effects’ reduction.	[175]
Peripheral nervous	Facial nerve regeneration	M	bFGF	Rat	Improved nerve axon maturation and increase of nerve regeneration rate.	[176]

**Table 9:** Clinical studies on empty gelatin-based DDSs.

Application	Outcome(s)	Ref.
Meningioma embolization	Greater penetration into the intra-tumoral vascular bed, reduction of intraoperative blood loss.	[179]
Rectal artery embolization for hemorrhoids	Low incidence of postoperative pain, low rate of ischemic complications.	[180]
Bone neoplasms’ embolization	Slowed tumor revascularization.	[181]
Uterine artery embolization for uterine fibroids	Pelvic pain and discomfort reduction of 92%, no severe complications.	[182]
	Resolution of 85% menorrhagia.	[183]
	Post-procedural pain comparable to PVA-based microparticles.	[184]
	Higher affinity to target the fibroid than PVA-based microparticles.	[185]
	Lower post-procedural pain and complications.	[186]
	Improved health-related quality of life and patient satisfaction.	[187]
	Greater degree of tumor infarction in patients treated with gelatin microspheres compared to patients treated with PVA-based microparticles.	[188]
	Efficacy comparable to PVA-based microparticles.	[189]
	Lower incidence of post-treatment tumor enlargement.	[190]
	Efficacy comparable to PVA-based microparticles.	[191]
	No significant reduction in pain or in the volume of administered narcotic.	[192]
	Similar pain scores and fentanyl dose of PVA-based particles. Less inflammatory response of PVA-based microparticles.	[193]

## Data Availability

No new data were created or analyzed in this study. Data sharing is not applicable to this article.

## References

[1] Liu D., Yang F., Xiong F., Gu N. (2016). The Smart Drug Delivery System and Its Clinical Potential. Theranostics.

[2] Adepu S., Ramakrishna S. (2021). Controlled Drug Delivery Systems: Current Status and Future Directions. Molecules.

[3] Langer R. (1998). Drug Delivery and Targeting. Nature.

[4] Jain K.K. (2020). Drug Delivery System. Methods in Molecular Biology.

[5] Hoffman A.S. (2008). The Origins and Evolution of “Controlled” Drug Delivery Systems. J. Control. Release.

[6] Langer R. (1990). New Methods of Drug Delivery. Science.

[7] Elzoghby A.O., Samy W.M., Elgindy N.A. (2012). Protein-Based Nanocarriers as Promising Drug and Gene Delivery Systems. J. Control. Release.

[8] Gorgieva S., Kokol V. (2011). Collagen-vs. Gelatine-Based Biomaterials and Their Biocompatibility: Review and Perspectives. Biomater. Appl. Nanomed..

[9] Pal A., Bajpai J., Bajpai A.K. (2018). Easy Fabrication and Characterization of Gelatin Nanocarriers and in Vitro Investigation of Swelling Controlled Release Dynamics of Paclitaxel. Polym. Bull..

[10] Tan H., Tu Z., Jia H., Gou X., Ngai T. (2018). Hierarchical Porous Protein Scaffold Templated from High Internal Phase Emulsion Costabilized by Gelatin and Gelatin Nanoparticles. Langmuir.

[11] Yang C., Wang J. (2014). Preparation and Characterization of Collagen Microspheres for Sustained Release of Steroidal Saponins. Mater. Res..

[12] Rossler B., Scherer D. (1995). Collagen Microparticles: Preparation and Properties. J. Microencapsul..

[13] Rathore P., Arora I., Rastogi S., Akhtar M., Singh S., Samim M. (2020). Collagen Nanoparticle-Mediated Brain Silymarin Delivery: An Approach for Treating Cerebral Ischemia and Reperfusion-Induced Brain Injury. Front. Neurosci..

[14] Seong Y.J., Song E.H., Park C., Lee H., Kang I.G., Kim H.E., Jeong S.H. (2020). Porous Calcium Phosphate–Collagen Composite Microspheres for Effective Growth Factor Delivery and Bone Tissue Regeneration. Mater. Sci. Eng. C..

[15] Calejo M.T., Almeida A.J., Fernandes A.I. (2012). Exploring a New Jellyfish Collagen in the Production of Microparticles for Protein Delivery. J. Microencapsul..

[16] Yeung P., Sin H.S., Chan S., Chan G.C.F., Chan B.P. (2015). Microencapsulation of Neuroblastoma Cells and Mesenchymal Stromal Cells in Collagen Microspheres: A 3D Model for Cancer Cell Niche Study. PLoS ONE.

[17] Kozlowska J., Stachowiak N., Prus W. (2019). Stability Studies of Collagen-Based Microspheres with Calendula Officinalis Flower Extract. Polym. Degrad. Stab..

[18] Zhang W., Wang X.-C., Wang J.-J., Zhang L.-l. (2019). Drugs Adsorption and Release Behavior of Collagen/Bacterial Cellulose Porous Microspheres. Int. J. Biol. Macromol..

[19] Zhang Z., Li X., Li Z., Bai Y., Liao G., Pan J., Zhang C. (2019). Collagen/Nano-Sized β-Tricalcium Phosphate Conduits Combined with Collagen Filaments and Nerve Growth Factor Promote Facial Nerve Regeneration in Miniature Swine: An in Vivo Study. Oral. Surg. Oral. Med. Oral. Pathol. Oral. Radiol..

[20] Berthold A., Cremer K., Rg Kreuter J. (1998). Collagen Microparticles: Carriers for Glucocorticosteroids. Eur. J. Pharm. Biopharm..

[21] Doi N., Jo J.I., Tabata Y. (2012). Preparation of Biodegradable Gelatin Nanospheres with a Narrow Size Distribution for Carrier of Cellular Internalization of Plasmid DNA. J. Biomater. Sci. Polym. Ed..

[22] Krause H.J., Rohdewald P. (1985). Preparation of gelatin nanocapsules and their pharmaceutical characterization. Pharm. Res..

[23] Zhou S., Li L., Chen C., Chen Y., Zhou L., Zhou F.H., Dong J., Wang L. (2021). Injectable Gelatin Microspheres Loaded with Platelet Rich Plasma Improve Wound Healing by Regulating Early Inflammation. Int. J. Med. Sci..

[24] Wang H., Boerman O.C., Sariibrahimoglu K., Li Y., Jansen J.A., Leeuwenburgh S.C.G. (2012). Comparison of Micro- vs. Nanostructured Colloidal Gelatin Gels for Sustained Delivery of Osteogenic Proteins: Bone Morphogenetic Protein-2 and Alkaline Phosphatase. Biomaterials.

[25] Leong W., Lau T.T., Wang D.A. (2013). A Temperature-Cured Dissolvable Gelatin Microsphere-Based Cell Carrier for Chondrocyte Delivery in a Hydrogel Scaffolding System. Acta Biomater..

[26] Kawadkar J., Jain R., Kishore R., Pathak A., Chauhan M.K. (2013). Formulation and Evaluation of Flurbiprofen-Loaded Genipin Cross-Linked Gelatin Microspheres for Intra-Articular Delivery. J. Drug. Target..

[27] Kudva A.K., Dikina A.D., Luyten F.P., Alsberg E., Patterson J. (2019). Gelatin Microspheres Releasing Transforming Growth Factor Drive in Vitro Chondrogenesis of Human Periosteum Derived Cells in Micromass Culture. Acta Biomater..

[28] Ramshaw J.A.M., Werkmeister J.A., Glattauer V. (1996). Collagen-Based Biomaterials. Biotechnol. Genet. Eng. Rev..

[29] Lee C.H., Singla A., Lee Y. (2001). Biomedical Applications of Collagen. Int. J. Pharm..

[30] Zwiorek K., Kloeckner J., Wagner E., Coester C. (2005). Gelatin nanoparticles as a new and simple gene delivery system. J. Pharm. Pharm. Sci..

[31] Lukin I., Erezuma I., Maeso L., Zarate J., Desimone M.F., Al-Tel T.H., Dolatshahi-Pirouz A., Orive G. (2022). Progress in Gelatin as Biomaterial for Tissue Engineering. Pharmaceutics.

[32] Johnston-Banks F.A. (1990). Gelatine. Food Gels.

[33] Patel Z.S., Yamamoto M., Ueda H., Tabata Y., Mikos A.G. (2008). Biodegradable Gelatin Microparticles as Delivery Systems for the Controlled Release of Bone Morphogenetic Protein-2. Acta Biomater..

[34] Samal S.K., Dash M., Van Vlierberghe S., Kaplan D.L., Chiellini E., van Blitterswijk C., Moroni L., Dubruel P. (2012). Cationic Polymers and Their Therapeutic Potential. Chem. Soc. Rev..

[35] Madkhali O., Mekhail G., Wettig S.D. (2019). Modified Gelatin Nanoparticles for Gene Delivery. Int. J. Pharm..

[36] Singh S., Rao R.K.V., Venugopal K., Manikandan R. (2002). Alteration in Dissolution Characteristic of Gelatin Containing Formulations: A Review of the Problem, Test Methods, and Solutions. Pharm. Technol..

[37] Taheri A., Abedian Kenari A.M., Gildberg A., Behnam S. (2009). Extraction and Physicochemical Characterization of Greater Lizardfish (Saurida Tumbil) Skin and Bone Gelatin. J. Food Sci..

[38] Zhou P., Regenstein J.M. (2006). Determination of Total Protein Content in Gelatin Solutions with the Lowry or Biuret Assay. J. Food Sci..

[39] Poppe J., Imeson A.P. (1997). Gelatin. Thickening and Gelling Agents for Food.

[40] Elzoghby A.O. (2013). Gelatin-Based Nanoparticles as Drug and Gene Delivery Systems: Reviewing Three Decades of Research. J. Control. Release.

[41] Babel W. (1996). Gelatine-Ein Vielseitiges Biopolymer. Tech. Chem..

[42] Haug I.J., Draget K.I., Smidsrød O. (2004). Physical and Rheological Properties of Fish Gelatin Compared to Mammalian Gelatin. Food Hydrocoll..

[43] Gudipati V. (2013). Fish Gelatin: A Versatile Ingredient for the Food and Pharmaceutical Industries. Marine Proteins and Peptides: Biological Activities and Aplications.

[44] Duthen S., Rochat C., Kleiber D., Violleau F., Daydé J., Raynaud C., Levasseur-Garcia C. (2018). Physicochemical characterization and study of molar mass of industrial gelatins by AsFlFFF-UV/MALS and chemometric approach. PLoS ONE.

[45] Gomez-Guillen M.C., Turnay J., Fernandez-Diaz M.D., Ulmo N., Lizarbe M.A., Montero P. (2002). Structural and physical properties of gelatin extracted from different marine species: A comparative study. Food Hydrocoll..

[46] Duconseille A., Astruc T., Quintana N., Meersman F., Sante-Lhoutellier V. (2015). Gelatin Structure and Composition Linked to Hard Capsule Dissolution: A Review. Food Hydrocoll..

[47] Guo L., Colby R.H., Lusignan C.P., Whitesides T.H. (2003). Kinetics of Triple Helix Formation in Semidilute Gelatin Solutions. Macromolecules.

[48] Karim A.A., Bhat R. (2009). Fish Gelatin: Properties, Challenges, and Prospects as an Alternative to Mammalian Gelatins. Food Hydrocoll..

[49] Wainewright F.W., Ward A.G., Courts A. (1977). Physical Tests for Gelatin and Gelatin Products. The Science and Technology of Gelatin.

[50] Schrieber R., Gareis H. (2007). Gelatine Handbook: Theory and Industrial Practice.

[51] Gomez-Guillen M.C., Gimenez B., Lopez-Caballero M.E., Montero M.P. (2011). Functional and Bioactive Properties of Collagen and Gelatin from Alternative Sources: A Review. Food Hydrocoll..

[52] Stainsby G., Ward A.G., Courts A. (1977). The Physical Chemistry of Gelatin in Solution. The Science and Technology of Gelatin.

[53] Alfaro A. (2015). da T.; Balbinot, E.; Weber, C.I.; Tonial, I.B.; Machado-Lunkes, A. Fish Gelatin: Characteristics, Functional Properties, Applications and Future Potentials. Food Eng. Rev..

[54] Michon C., Cuvelier G., Relkin P., Launay B. (1997). Influence of Thermal History on the Stability of Gelatin Gels. Int. J. Biol. Macromol..

[55] Borchard W., Burg B. (2007). Molecular mechanisms during the thermoreversible gelation of gelatin-water-systems. Interfaces Condens. Syst..

[56] Wang R., Hartel R.W. (2022). Confectionery Gels: Gelling Behavior and Gel Properties of Gelatin in Concentrated Sugar Solutions. Food Hydrocoll..

[57] Alipal J., Mohd Pu’ad N.A.S., Lee T.C., Nayan N.H.M., Sahari N., Basri H., Idris M.I., Abdullah H.Z. (2021). A Review of Gelatin: Properties, Sources, Process, Applications, and Commercialisation. Mater. Today Proc..

[58] Mahmoudi Saber M. (2019). Strategies for surface modification of gelatin-based nanoparticles. Colloids Surf. B. Biointerfaces.

[59] Su K., Wang C. (2015). Recent Advances in the Use of Gelatin in Biomedical Research. Biotechnol. Lett..

[60] Karim A.A., Bhat R. (2008). Gelatin Alternatives for the Food Industry: Recent Developments, Challenges and Prospects. Trends Food Sci. Technol..

[61] Igoe R.S. (1983). Dictionary of Food Ingredients.

[62] Clark A.H., Ross-Murphy S.B. (1987). Structural and Mechanical Properties of Biopolymer Gels. Adv. Polym. Sci..

[63] Sionkowska A., Skrzyński S., Śmiechowski K., Kołodziejczak A. (2016). The Review of Versatile Application of Collagen. Polym. Adv. Technol..

[64] Elgadir M.A., Mirghani M.E.S., Adam A. (2013). Fish gelatin and its applications in selected pharmaceutical aspects as alternative source to pork gelatin. J. Food Agric. Environ..

[65] Fan J., Zhuang Y., Li B. (2013). Effects of Collagen and Collagen Hydrolysate from Jellyfish Umbrella on Histological and Immunity Changes of Mice Photoaging. Nutrients.

[66] Young S., Wong M., Tabata Y., Mikos A.G. (2005). Gelatin as a Delivery Vehicle for the Controlled Release of Bioactive Molecules. J. Control. Release.

[67] Lee E.J., Lim K.H. (2017). Hardly Water-Soluble Drug-Loaded Gelatin Nanoparticles Sustaining a Slow Release: Preparation by Novel Single-Step O/W/O Emulsion Accompanying Solvent Diffusion. Bioprocess. Biosyst. Eng..

[68] Nur Hanani Z.A., Roos Y.H., Kerry J.P. (2014). Use and Application of Gelatin as Potential Biodegradable Packaging Materials for Food Products. Int. J. Biol. Macromol..

[69] Weiss A.V., Fischer T., Iturri J., Benitez R., Toca-Herrera J.L., Schneider M. (2019). Mechanical Properties of Gelatin Nanoparticles in Dependency of Crosslinking Time and Storage. Colloids Surf. B. Biointerfaces.

[70] Hathout R.M., Omran M.K. (2016). Gelatin-Based Particulate Systems in Ocular Drug Delivery. Pharm. Dev. Technol..

[71] Slomkowski S., Alemán J.V., Gilbert R.G., Hess M., Horie K., Jones R.G., Kubisa P., Meisel I., Mormann W., Penczek S. (2011). Terminology of Polymers and Polymerization Processes in Dispersed Systems (IUPAC Recommendations 2011). Pure Appl. Chem..

[72] Oliveira M.B., Mano J.F. (2011). Polymer-Based Microparticles in Tissue Engineering and Regenerative Medicine. Biotechnol. Prog..

[73] Letícia Braz A., Ahmed I. (2017). Manufacturing Processes for Polymeric Micro and Nanoparticles and Their Biomedical Applications. AIMS Bioeng..

[74] Stevanović M. (2017). Polymeric Micro- and Nanoparticles for Controlled and Targeted Drug Delivery. Nanostructures for Drug Delivery.

[75] Bruschi L.M. (2015). Drug Delivery Systems. Strategies to Modify the Drug. Release from Pharmaceutical Systems.

[76] Azarmi S., Huang Y., Chen H., Mcquarrie S., Abrams D., Roa W., Finlay W.H., Miller G.G., Löbenberg R. (2006). Optimization of a Two-Step Desolvation Method for Preparing Gelatin Nanoparticles and Cell Uptake Studies in 143B Osteosarcoma Cancer Cells. J. Pharm. Pharm. Sci..

[77] Nejat H., Rabiee M., Varshochian R., Tahriri M., Jazayeri H.E., Rajadas J., Ye H., Cui Z., Tayebi L. (2017). Preparation and Characterization of Cardamom Extract-Loaded Gelatin Nanoparticles as Effective Targeted Drug Delivery System to Treat Glioblastoma. React. Funct. Polym..

[78] Nahar M., Mishra D., Dubey V., Jain N.K. (2008). Development, Characterization, and Toxicity Evaluation of Amphotericin B-Loaded Gelatin Nanoparticles. Nanomedicine.

[79] Hathout R.M., Metwally A.A. (2019). Gelatin Nanoparticles. Methods Mol. Biol..

[80] Koletti A.E., Tsarouchi E., Kapourani A., Kontogiannopoulos K.N., Assimopoulou A.N., Barmpalexis P. (2020). Gelatin Nanoparticles for NSAID Systemic Administration: Quality by Design and Artificial Neural Networks Implementation. Int. J. Pharm..

[81] Kaur A., Jain S., Tiwary A.K. (2008). Mannan-Coated Gelatin Nanoparticles for Sustained and Targeted Delivery of Didanosine: In Vitro and in Vivo Evaluation. Acta Pharm..

[82] Pande V. (2015). Studies on the Characteristics of Zaltoprofen Loaded Gelatin Nanoparticles by Nanoprecipitation. Inven. Rapid NDDS.

[83] Sharifi F., Hadizadeh F., Sadeghi F., Hamed Mosavian M.T., Zarei C. (2016). Process Optimization, Physical Properties, and Environmental Stability of an α-Tocopherol Nanocapsule Preparation Using Complex Coacervation Method and Full Factorial Design. Chem. Eng. Commun..

[84] Singh V., Chaudhary A.K. (2010). Development and characterization of Rosiglitazone loaded gelatin nanoparticles using two step desolvation method. Int. J. Pharm. Sci. Rev. Res..

[85] Ogilvie-Battersby J.D., Nagarajan R., Mosurkal R., Orbey N. (2022). Microencapsulation and Controlled Release of Insect Repellent Geraniol in Gelatin/Gum Arabic Microcapsules. Colloids Surf. Physicochem. Eng. Asp..

[86] Ahmed M.A., Al-Kahtani H.A., Jaswir I., AbuTarboush H., Ismail E.A. (2020). Extraction and Characterization of Gelatin from Camel Skin (Potential Halal Gelatin) and Production of Gelatin Nanoparticles. Saudi J. Biol. Sci..

[87] Zhao L., Mustapha O., Shafique S., Jamshaid T., Din F.U., Mehmood Y., Anwer K., Yousafi Q.U.A., Hussain T., Khan I.U. (2020). Electrospun Gelatin Nanocontainers for Enhanced Biopharmaceutical Performance of Piroxicam: In Vivo and in Vitro Investigations. Int. J. Nanomed..

[88] Wu H., Zhang Z.X., Zhao H.P., Wu D.C., Wu B.L., Cong R. (2004). Preparation of Sodium Fluoride-Loaded Gelatin Microspheres, Characterization and Cariostatic Studies. J. Microencapsul..

[89] Loepfe M., Duss A., Zafeiropoulou K.A., Björgvinsdóttir O., D’Este M., Eglin D., Fortunato G., Klasen J., Ferguson S.J., Wuertz-Kozak K. (2019). Electrospray-Based Microencapsulation of Epigallocatechin 3-Gallate for Local Delivery into the Intervertebral Disc. Pharmaceutics.

[90] Naskar S., Sharma S., Kuotsu K. (2019). A Smart Gelatin Nanoparticle for Delivery of Metoprolol Succinate: A Strategy for Enhancing the Therapeutic Efficacy by Improving Bioavailability. J. Drug. Deliv. Sci. Technol..

[91] Turner P.A., Thiele J.S., Stegemann J.P. (2017). Growth Factor Sequestration and Enzyme-Mediated Release from Genipin-Crosslinked Gelatin Microspheres. J. Biomater. Sci. Polym. Ed..

[92] Kocer Z., Aru B., Sezer U.A., Demirel G.Y., Beker U., Sezer S. (2019). Process Optimisation, Biocompatibility and Anti-Cancer Efficacy of Curcumin Loaded Gelatine Microparticles Cross-Linked with Dialdeyhde Carboxymethyl Cellulose. J. Microencapsul..

[93] Contessi Negrini N., Lipreri M.V., Tanzi M.C., Farè S. (2020). In Vitro Cell Delivery by Gelatin Microspheres Prepared in Water-in-Oil Emulsion. J. Mater. Sci. Mater. Med..

[94] Shimokawa K.I., Saegusa K., Wada Y., Ishii F. (2013). Physicochemical Properties and Controlled Drug Release of Microcapsules Prepared by Simple Coacervation. Colloids Surf. Biointerfaces.

[95] Baseer A., Koenneke A., Zapp J., Khan S.A., Schneider M. (2019). Design and Characterization of Surface-Crosslinked Gelatin Nanoparticles for the Delivery of Hydrophilic Macromolecular Drugs. Macromol. Chem. Phys..

[96] Xu Y., Peng J., Richards G., Lu S., Eglin D. (2019). Optimization of Electrospray Fabrication of Stem Cell–Embedded Alginate–Gelatin Microspheres and Their Assembly in 3D-Printed Poly(ε-Caprolactone) Scaffold for Cartilage Tissue Engineering. J. Orthop. Translat..

[97] Liao S., Meng H., Zhao J., Lin W., Liu X., Tian Z., Lan L., Yang H., Zou Y., Xu Y. (2022). Injectable Adipose-Derived Stem Cells-Embedded Alginate-Gelatin Microspheres Prepared by Electrospray for Cartilage Tissue Regeneration. J. Orthop. Translat..

[98] Nguyen A.H., McKinney J., Miller T., Bongiorno T., McDevitt T.C. (2015). Gelatin Methacrylate Microspheres for Controlled Growth Factor Release. Acta Biomater..

[99] Santos M.B., de Carvalho C.W.P., Garcia-Rojas E.E. (2021). Microencapsulation of Vitamin D3 by Complex Coacervation Using Carboxymethyl Tara Gum (Caesalpinia Spinosa) and Gelatin A. Food Chem..

[100] Wang H., Zou Q., Boerman O.C., Nijhuis A.W.G., Jansen J.A., Li Y., Leeuwenburgh S.C.G. (2013). Combined Delivery of BMP-2 and BFGF from Nanostructured Colloidal Gelatin Gels and Its Effect on Bone Regeneration in Vivo. J. Control. Release.

[101] Lam P.L., Kok S.H.L., Ho Y.W., Wong R.S.M., Cheng G.Y.M., Cheng C.H., Lam K.H., Gambari R., Lee K.K.H., Chui C.H. (2013). A Novel Green Gelatin-Agar Microencapsulation System with P. Urinaria as an Improved Anti-A. Niger Model. Carbohydr. Polym..

[102] Quiroz-Reyes C.N., Ronquillo-De Jesús E., Duran-Caballero N.E., Aguilar-Méndez M.Á. (2014). Development and Characterization of Gelatin Nanoparticles Loaded with a Cocoa-Derived Polyphenolic Extract. Fruits.

[103] Xing F., Cheng G., Yi K., Ma L. (2005). Nanoencapsulation of Capsaicin by Complex Coacervation of Gelatin, Acacia, and Tannins. J. Appl. Polym. Sci..

[104] Yao R., Zhang R., Luan J., Lin F. (2012). Alginate and Alginate/Gelatin Microspheres for Human Adipose-Derived Stem Cell Encapsulation and Differentiation. Biofabrication.

[105] Narayanan D., Geena M.G., Lakshmi H., Koyakutty M., Nair S., Menon D. (2013). Poly-(Ethylene Glycol) Modified Gelatin Nanoparticles for Sustained Delivery of the Anti-Inflammatory Drug Ibuprofen-Sodium: An in Vitro and in Vivo Analysis. Nanomedicine.

[106] Mahor A., Prajapati S.K., Verma A., Gupta R., Iyer A.K., Kesharwani P. (2016). Moxifloxacin Loaded Gelatin Nanoparticles for Ocular Delivery: Formulation and in-Vitro, in-Vivo Evaluation. J. Colloid. Interface Sci..

[107] Shokry M., Hathout R.M., Mansour S. (2018). Exploring Gelatin Nanoparticles as Novel Nanocarriers for Timolol Maleate: Augmented in-Vivo Efficacy and Safe Histological Profile. Int. J. Pharm..

[108] Coester C.J., Langer K., Von Briesen H., Kreuter J. (2000). Gelatin nanoparticles by two step desolvation- a new preparation method, surface modifications and cell uptake. J. Microencapsul..

[109] de Oliveira C.A., Peres D.D.A., Graziola F., Chacra N.A.B., de Araújo G.L.B., Flórido A.C., Mota J., Rosado C., Velasco M.V.R., Rodrigues L.M. (2016). Cutaneous Biocompatible Rutin-Loaded Gelatin-Based Nanoparticles Increase the SPF of the Association of UVA and UVB Filters. Eur. J. Pharm. Sci..

[110] Azimi B., Nourpanah P., Rabiee M., Arbab S. (2013). Producing Gelatin Nanoparticles as Delivery System for Bovine Serum Albumin. Iran. Biomed. J..

[111] Subara D., Jaswir I., Alkhatib, Noorbatcha I.A.N. (2017). Process Optimization for the Production of Fish Gelatin Nanoparticles. Int. Food Res. J..

[112] Khan S.A., Schneider M. (2014). Stabilization of Gelatin Nanoparticles without Crosslinking. Macromol. Biosci..

[113] Lee E.J., Khan S.A., Lim K.H. (2011). Gelatin Nanoparticle Preparation by Nanoprecipitation. J. Biomater. Sci. Polym. Ed..

[114] Khan S.A., Schneider M. (2013). Improvement of Nanoprecipitation Technique for Preparation of Gelatin Nanoparticles and Potential Macromolecular Drug Loading. Macromol. Biosci..

[115] Fathollahipour S., Abouei Mehrizi A., Ghaee A. Fabrication and Characterization of Gelatin Nanoparticles by Nanoprecipitation as a Delivery System for Erythromycin. Proceedings of the 11th International Seminar on Polymer Science and Technology.

[116] Wang J., Chen S.H., Xu Z.C. (2008). Synthesis and Properties Research on the Nanocapsulated Capsaicin by Simple Coacervation Method. J. Dispers. Sci. Technol..

[117] Zhang J., Jia G., Wanbin Z., Minghao J., Wei Y., Hao J., Liu X., Gan Z., Sun A. (2021). Nanoencapsulation of Zeaxanthin Extracted from Lycium Barbarum L. by Complex Coacervation with Gelatin and CMC. Food Hydrocoll..

[118] Lam P.L., Lee K.K.H., Kok S.H.L., Cheng G.Y.M., Tao X.M., Hau D.K.P., Yuen M.C.W., Lam K.H., Gambari R., Chui C.H. (2012). Development of Formaldehyde-Free Agar/Gelatin Microcapsules Containing Berberine HCl and Gallic Acid and Their Topical and Oral Applications. Soft Matter.

[119] Li L., Au W., Hua T., Zhao D., Wong K. (2013). Improvement in Antibacterial Activity of Moxa Oil Containing Gelatin-Arabic Gum Microcapsules. Text. Res. J..

[120] Esfahani R., Jafari S.M., Jafarpour A., Dehnad D. (2019). Loading of Fish Oil into Nanocarriers Prepared through Gelatin-Gum Arabic Complexation. Food Hydrocoll..

[121] Yoshioka T., Hashida M., Muranishi S., Sezaki H. (1981). Specific delivery of mitomycin c to the liver spleen and lung: Nano- and microspherical carriers of gelatin. Int. J. Pharm..

[122] Tabata Y., Hijikata S., Muniruzzaman M., Ikadaf Y. (1999). Neovascularization Effect of Biodegradable Gelatin Microspheres Incorporating Basic Fibroblast Growth Factor. J. Biomater. Sci. Polym. Ed..

[123] Zhang S., Dai W., Lu Z., Lei Z., Yang B., He B., Zhou H., Cao J. (2018). Preparation and Evaluation of Cefquinome-Loaded Gelatin Microspheres and the Pharmacokinetics in Pigs. J. Vet. Pharm..

[124] Chen H., Xing X., Tan H., Jia Y., Zhou T., Chen Y., Ling Z., Hu X. (2017). Covalently Antibacterial Alginate-Chitosan Hydrogel Dressing Integrated Gelatin Microspheres Containing Tetracycline Hydrochloride for Wound Healing. Mater. Sci. Eng. C..

[125] Wang J., Tauchi Y., Deguchi Y., Morimoto K., Tabata Y., Ikada Y. (2000). Positively Charged Gelatin Microspheres as Gastric Mucoadhesive Drug Delivery System for Eradication of H. Pylori. Drug. Deliv. J. Deliv. Target. Ther. Agents.

[126] Li A.K., Wu X.S. (1998). Gelatin Nanoencapsulation of Protein/Peptide Drugs Using an Emulsifier-Free Emulsion Method. J. Microencapsul..

[127] Cascone M.G., Lazzeri L. (2002). Gelatin Nanoparticles Produced by a Simple W/O Emulsion as Delivery System for Methotrexate. J. Mater. Sci. Mater. Med..

[128] Houshyari A., Heydari M., Bagheri M., Nezafati N. (2018). Preparation of Gelatin Nanoparticles by a Water-in-Oil Emulsion Method for Water-Soluble Model Drug Encapsulation. Mater. Today Proc..

[129] Kim J.S., Park J.H., Jeong S.C., Kim D.S., Yousaf A.M., Din F.U., Kim J.O., Yong C.S., Youn Y.S., Oh K.T. (2018). Novel Revaprazan-Loaded Gelatin Microsphere with Enhanced Drug Solubility and Oral Bioavailability. J. Microencapsul..

[130] Li D.X., Kim J.O., Oh D.H., Lee W.S., Hong M.J., Kang J.Y., Choi J.S., Woo J.S., Yong C.S., Choi H.G. (2009). Development of Nifedipine-Loaded Coated Gelatin Microcapsule as a Long Acting Oral Delivery. Arch. Pharm. Res..

[131] Li D.X., Yan Y.D., Oh D.H., Yang K.Y., Seo Y.G., Kim J.O., Kim Y.I., Yong C.S., Choi H.G. (2010). Development of Valsartan-Loaded Gelatin Microcapsule without Crystal Change Using Hydroxypropylmethylcellulose as a Stabilizer. Drug. Deliv..

[132] Yousaf A.M., Kim D.W., Kim J.K., Kim J.O., Yong C.S., Choi H.G. (2015). Novel Fenofibrate-Loaded Gelatin Microcapsules with Enhanced Solubility and Excellent Flowability: Preparation and Physicochemical Characterization. Powder Technol..

[133] Li D.X., Oh Y.K., Lim S.J., Kim J.O., Yang H.J., Sung J.H., Yong C.S., Choi H.G. (2008). Novel Gelatin Microcapsule with Bioavailability Enhancement of Ibuprofen Using Spray-Drying Technique. Int. J. Pharm..

[134] Yong C.-S., Li D.-X., Oh D.-H., Kim J.-A., Yoo B.-K., Woo J.-S., Rhee J.-D., Choi H.-G. (2006). Retarded Dissolution of Ibuprofen in Gelatin Microcapsule by Cross-Linking with Glutaradehyde. Arch. Pharm. Res..

[135] Piao M.G., Yang C.W., Li D.X., Kim J.O., Jang K.-Y., Yoo B.K., Kim J.A., Woo J.S., Lyoo W.S., Han S.S. (2008). Preparation and in vivo evaluation of piroxicam-loaded gelatin microcapsule by spray drying technique. Biol. Pharm. Bull..

[136] Hani N., Azarian M.H., Torkamani A.E., Kamil Mahmood W.A. (2016). Characterisation of Gelatin Nanoparticles Encapsulated with Moringa Oleifera Bioactive Extract. Int. J. Food Sci. Technol..

[137] Torkamani A.E., Syahariza Z.A., Norziah M.H., Mahmood W.A.K., Juliano P. (2018). Production and Characterization of Gelatin Spherical Particles Formed via Electrospraying and Encapsulated with Polyphenolic Antioxidants from Momordica Charantia. Food Bioproc. Tech..

[138] Fessi C., Devissaguet J.P., Puisieux F., Thies C. (1990). Process for the Preparation of Dispersible Colloidal Systems of a Substance in the Form of Nanoparticles. U.S. Patent.

[139] Minost A., Delaveau J., Bolzinger M.A., Fessi H., Elaissari A.H. (2012). Nanoparticles via Nanoprecipitation Process. Recent. Pat. Drug. Deliv. Formul..

[140] Martínez Rivas C.J., Tarhini M., Badri W., Miladi K., Greige-Gerges H., Nazari Q.A., Galindo Rodríguez S.A., Román R.Á., Fessi H., Elaissari A. (2017). Nanoprecipitation Process: From Encapsulation to Drug Delivery. Int. J. Pharm..

[141] Kruyt H.R., Bungenberg de Jong H.G. (1929). Koazervation. Proc. K. Ned. Akad. Wet..

[142] Nairm J.G. (1995). Coacervation-phase separation Technology. Adv. Pharm. Sci..

[143] Phares R., Sperandio G.J. (1964). Coating Pharmaceuticals by Coacervation. J. Pharm. Sci..

[144] Arshady R. (1990). Microspheres and Microcapsules, a Survey of Manufacturing Techniques Part II: Coacervation. Polym. Eng. Sci..

[145] Swider E., Koshkina O., Tel J., Cruz L.J., de Vries I.J.M., Srinivas M. (2018). Customizing Poly(Lactic-Co-Glycolic Acid) Particles for Biomedical Applications. Acta Biomater..

[146] Everett D.H. (1972). Manual of Symbols and Terminology for Physicochemical Quantities and Units, Appendix II: Definitions, Terminology and Symbols in Colloid and Surface Chemistry. Pure Appl. Chem..

[147] Khan A.Y., Talegaonkar S., Iqbal Z., Ahmed J., Krishan Khar R. (2006). Multiple Emulsions: An Overview. Curr. Drug. Deliv..

[148] Schramm L.L. (2005). Applied surfactants Principles and Applications. Emulsions, Foams, and Suspensions.

[149] Percy S.R. (1872). Improvement in Drying and Concentrating Liquid Substances by Atomizing. U.S. Patent.

[150] Cal K., Sollohub K. (2010). Spray Drying Technique. I: Hardware and Process Parameters. J. Pharm. Sci..

[151] Santos D., Maurício A.C., Sencadas V., Santos J.D., Fernandes M.H., Gomes P.S. (2017). Spray Drying: An Overview. Biomaterials-Physics and Chemistry–New Edition.

[152] Morais A.Í.S., Vieira E.G., Afewerki S., Sousa R.B., Honorio L.M.C., Cambrussi A.N.C.O., Santos J.A., Bezerra R.D.S., Furtini J.A.O., Silva-Filho E.C. (2020). Fabrication of Polymeric Microparticles by Electrospray: The Impact of Experimental Parameters. J. Funct. Biomater..

[153] Taylor I.G. (1964). Disintegration of Water Drops in an Electric Field. Proc. R. Soc. Lond. A Math. Phys. Sci..

[154] Lord Rayleigh F.R.S. (2009). On the equilibrium of liquid conducting masses charged with electricity. Lond. Edinb. Dublin Philos. Mag. J. Sci..

[155] Xie J., Lim L.K., Phua Y., Hua J., Wang C.H. (2006). Electrohydrodynamic Atomization for Biodegradable Polymeric Particle Production. J. Colloid. Interface Sci..

[156] Bock N., Woodruff M.A., Hutmacher D.W., Dargaville T.R. (2011). Electrospraying, a Reproducible Method for Production of Polymeric Microspheres for Biomedical Applications. Polymers.

[157] Vonnegut B., Neubauer R.L., Geller M., Maynard K. (1952). Production of monodisperse liquid particles by electrical atomization. J. Colloid. Sci..

[158] Tapia-Hernández J.A., Torres-Chávez P.I., Ramírez-Wong B., Rascón-Chu A., Plascencia-Jatomea M., Barreras-Urbina C.G., Rangel-Vázquez N.A., Rodríguez-Félix F. (2015). Micro- and Nanoparticles by Electrospray: Advances and Applications in Foods. J. Agric. Food Chem..

[159] Oe S., Fukunaka Y., Hirose T., Yamaoka Y., Tabata Y. (2003). A Trial on Regeneration Therapy of Rat Liver Cirrhosis by Controlled Release of Hepatocyte Growth Factor. J. Control. Release.

[160] Nitta N., Sonoda A., Seko A., Ohta S., Nagatani Y., Tsuchiya K., Otani H., Tanaka T., Kanasaki S., Takahashi M. (2010). Combination of Cisplatin-Eluting Gelatin Microspheres and Flavopiridol Enhances Anti-Tumour Effects in a Rabbit VX2 Liver Tumour Model. Br. J. Radiol..

[161] Ohta S., Nitta N., Sonoda A., Seko A., Tanaka T., Takahashi M., Takemura S., Tabata Y., Murata K. (2009). Prolonged Local Persistence of Cisplatin-Loaded Gelatin Microspheres and Their Chemoembolic Anti-Cancer Effect in Rabbits. Eur. J. Radiol..

[162] Gunji S., Obama K., Matsui M., Tabata Y., Sakai Y. (2013). A Novel Drug Delivery System of Intraperitoneal Chemotherapy for Peritoneal Carcinomatosis Using Gelatin Microspheres Incorporating Cisplatin. Surgery.

[163] Obata Y., Nishino T., Kushibiki T., Tomoshige R., Xia Z., Miyazaki M., Abe K., Koji T., Tabata Y., Kohno S. (2012). HSP47 SiRNA Conjugated with Cationized Gelatin Microspheres Suppresses Peritoneal Fibrosis in Mice. Acta Biomater..

[164] Singh A., Xu J., Mattheolabakis G., Amiji M. (2016). EGFR-Targeted Gelatin Nanoparticles for Systemic Administration of Gemcitabine in an Orthotopic Pancreatic Cancer Model. Nanomedicine.

[165] Kushibiki T., Matsumoto K., Nakamura T., Tabata Y. (2004). Suppression of the progress of disseminated pancreatic cancer cells by NK4 plasmid DNA released from cationized gelatin microspheres. Pharm. Res..

[166] Hirose F., Kiryu J., Tabata Y., Tamura H., Musashi K., Takase N., Usui H., Kuwayama S., Kato A., Yoshimura N. (2018). Experimental Proliferative Vitreoretinopathy in Rabbits by Delivery of Bioactive Proteins with Gelatin Microspheres. Eur. J. Pharm. Biopharm..

[167] Chuang Y.L., Fang H.W., Ajitsaria A., Chen K.H., Su C.Y., Liu G.S., Tseng C.L. (2019). Development of Kaempferol-Loaded Gelatin Nanoparticles for the Treatment of Corneal Neovascularization in Mice. Pharmaceutics.

[168] Luo Y., Li D., Xie X., Kang P.D. (2019). Porous, Lithium-Doped Calcium Polyphosphate Composite Scaffolds Containing Vascular Endothelial Growth Factor (VEGF)-Loaded Gelatin Microspheres for Treating Glucocorticoid-Induced Osteonecrosis of the Femoral Head. Biomed. Mater..

[169] Saravanan M., Bhaskar K., Maharajan G., Pillai K.S. (2004). Ultrasonically Controlled Release and Targeted Delivery of Diclofenac Sodium via Gelatin Magnetic Microspheres. Int. J. Pharm..

[170] Kumar R., Nagarwal R.C., Dhanawat M., Pandit J.K. (2011). In-Vitro and In-Vivo Study of Indomethacin Loaded Gelatin Nanoparticles. J. Biomed. Nanotechnol..

[171] Lu Z., Yeh T.-K., Tsai M., Jessie L.-S., Au J., Guill Wientjes M. (2004). Paclitaxel-Loaded Gelatin Nanoparticles for Intravesical Bladder Cancer Therapy. Clin. Cancer Res..

[172] De Clercq K., Xie F., de Wever O., Descamps B., Hoorens A., Vermeulen A., Ceelen W., Vervaet C. (2019). Preclinical Evaluation of Local Prolonged Release of Paclitaxel from Gelatin Microspheres for the Prevention of Recurrence of Peritoneal Carcinomatosis in Advanced Ovarian Cancer. Sci. Rep..

[173] Tseng C.L., Su W.Y., Yen K.C., Yang K.C., Lin F.H. (2009). The Use of Biotinylated-EGF-Modified Gelatin Nanoparticle Carrier to Enhance Cisplatin Accumulation in Cancerous Lungs via Inhalation. Biomaterials.

[174] Kaul G., Amiji M. (2005). Tumor-Targeted Gene Delivery Using Poly(Ethylene Glycol)-Modified Gelatin Nanoparticles: In Vitro and In Vivo Studies. Pharm. Res..

[175] Nakase H., Okazaki K., Tabata Y., Ozeki M., Watanabe N., Ohana M., Uose S., Uchida K., Nishi T., Mastuura M. (2002). New cytokine delivery system using gelatin microspheres containing interleukin-10 for experimental inflammatory bowel disease. J. Pharm. Exp..

[176] Matsumine H., Sasaki R., Tabata Y., Matsui M., Yamato M., Okano T., Sakurai H. (2016). Facial Nerve Regeneration Using Basic Fibroblast Growth Factor-Impregnated Gelatin Microspheres in a Rat Model. J. Tissue Eng. Regen. Med..

[177] Ramamoorth M., Narvekar A. (2015). Non Viral Vectors in Gene Therapy—An Overview. J. Clin. Diagn. Res..

[178] My Beaujeux R., Laurent A., Wassef M., Casasco A., Gobin Y.P., Aymard A., Rüfenacht D., Merland J.J. (1996). Trisacryl Gelatin Microspheres for Therapeutic Embolization, II: Preliminary Clinical Evaluation in Tumors and Arteriovenous Malformations. AJNR Am. J. Neuroradiol..

[179] Bendszus M., Klein R., Burger R., Warmuth-Metz M., Hofmann E., Solymosi L. (2000). Efficacy of Trisacryl Gelatin Microspheres versus Polyvinyl Alcohol Particles in the Preoperative Embolization of Meningiomas. AJNR Am. J. Neuroradiol..

[180] Küçükay M.B., Küçükay F. (2021). Superior Rectal Artery Embolization with Tris-Acryl Gelatin Microspheres: A Randomized Comparison of Particle Size. J. Vasc. Interv. Radiol..

[181] Basile A., Rand T., Lomoschitz F., Toma C., Lupattelli T., Kettenbach J., Lammer J. (2004). Trisacryl Gelatin Microspheres versus Polyvinyl Alcohol Particles in the Preoperative Embolization of Bone Neoplasms. Cardiovasc. Interv. Radiol..

[182] Spies J.B., Benenati J.F., Worthington-Kirsch R.L., Pelage J.P. (2001). Initial Experience with Use of Tris-Acryl Gelatin Microspheres for Uterine Artery Embolization for Leiomyomata. J. Vasc. Interv. Radiol..

[183] Pelage J.P., le Dref O., Beregi J.P., Nonent M., Robert Y., Cosson M., Jacob D., Truc J.B., Laurent A., Rymer R. (2003). Limited Uterine Artery Embolization with Tris-Acryl Gelatin Microspheres for Uterine Fibroids. J. Vasc. Interv. Radiol..

[184] Ryu R.K., Omary R.A., Sichlau M.J., Siddiqi A., Chrisman H.B., Nemcek A.A., Vogelzang R.L. (2003). Comparison of Pain after Uterine Artery Embolization Using Tris-Acryl Gelatin Microspheres versus Polyvinyl Alcohol Particles. Cardiovasc. Interv. Radiol..

[185] Chua G.C., Wilsher M., Young M.P.A., Manyonda I., Morgan R., Belli A.M. (2005). Comparison of Particle Penetration with Non-Spherical Polyvinyl Alcohol versus Trisacryl Gelatin Microspheres in Women Undergoing Premyomectomy Uterine Artery Embolization. Clin. Radiol..

[186] Joffre F., Tubiana J.M., Pelage J.P. (2004). FEMIC (Fibromes Embolisés Aux MICrosphères Calibrées): Uterine Fibroid Embolization Using Tris-Acryl Microspheres. A French Multicenter Study. Cardiovasc. Interv. Radiol..

[187] Spies J.B., Cornell C., Worthington-Kirsch R., Lipman J.C., Benenati J.F. (2007). Long-Term Outcome from Uterine Fibroid Embolization with Tris-Acryl Gelatin Microspheres: Results of a Multicenter Study. J. Vasc. Interv. Radiol..

[188] Siskin G.P., Beck A., Schuster M., Mandato K., Englander M., Herr A. (2008). Leiomyoma Infarction after Uterine Artery Embolization: A Prospective Randomized Study Comparing Tris-Acryl Gelatin Microspheres versus Polyvinyl Alcohol Microspheres. J. Vasc. Interv. Radiol..

[189] Worthington-Kirsch R.L., Siskin G.P., Hegener P., Chesnick R. (2011). Comparison of the Efficacy of the Embolic Agents Acrylamido Polyvinyl Alcohol Microspheres and Tris-Acryl Gelatin Microspheres for Uterine Artery Embolization for Leiomyomas: A Prospective Randomized Controlled Trial. Cardiovasc. Interv. Radiol..

[190] Yu S.C.H., Lok I., Ho S.S.Y., Tong M.M.B., Hui J.W.Y. (2011). Comparison of Clinical Outcomes of Tris-Acryl Microspheres versus Polyvinyl Alcohol Microspheres for Uterine Artery Embolization for Leiomyomas: Results of a Randomized Trial. J. Vasc. Interv. Radiol..

[191] Shlansky-Goldberg R.D., Rosen M.A., Mondschein J.I., Stavropoulos S.W., Trerotola S.O., Diaz-Cartelle J. (2014). Comparison of Polyvinyl Alcohol Microspheres and Tris-Acryl Gelatin Microspheres for Uterine Fibroid Embolization: Results of a Single-Center Randomized Study. J. Vasc. Interv. Radiol..

[192] Katsumori T., Miura H., Yoshikawa T., Seri S., Kotera Y., Asato A. (2020). Intra-Arterial Lidocaine Administration for Anesthesia after Uterine Artery Embolization with Trisacryl Gelatin Microspheres for Leiomyoma. J. Vasc. Interv. Radiol..

[193] Han K., Kim S.Y., Kim H.J., Kwon J.H., Kim G.M., Lee J., Won J.Y., Shin H.J., Yoon E.J., Kim M.D. (2021). Nonspherical Polyvinyl Alcohol Particles versus Tris-Acryl Microspheres: Randomized Controlled Trial Comparing Pain after Uterine Artery Embolization for Symptomatic Fibroids. Radiology.

[194] Nitta N., Ohta S., Tanaka T., Takazakura R., Toyama T., Sonoda A., Seko A., Furukawa A., Takahashi M., Murata K. (2009). An Initial Clinical Study on the Efficacy of Cisplatin-Releasing Gelatin Microspheres for Metastatic Liver Tumors. Eur. J. Radiol..

[195] Toyama T., Nitta N., Ohta S., Tanaka T., Nagatani Y., Takahashi M., Murata K., Shiomi H., Naka S., Kurumi Y. (2012). Clinical Trial of Cisplatin-Conjugated Gelatin Microspheres for Patients with Hepatocellular Carcinoma. Jpn. J. Radiol..

[196] Marui A., Tabata Y., Kojima S., Yamamoto M., Tambara K., Nishina T., Saji Y., Inui K.-i., Hashida T., Yokoyama S. (2007). A novel approach to therapeutic angiogenesis for patients with critical limb ischemia by sustained release of basic fibroblast growth factor using biodegradable gelatin hydrogel: An initial report of the phase I-IIa study. Circ. J..

[197] Kumagai M., Marui A., Tabata Y., Takeda T., Yamamoto M., Yonezawa A., Tanaka S., Yanagi S., Ito-Ihara T., Ikeda T. (2016). Safety and Efficacy of Sustained Release of Basic Fibroblast Growth Factor Using Gelatin Hydrogel in Patients with Critical Limb Ischemia. Heart Vessel..

[198] Hashimoto T., Koyama H., Miyata T., Hosaka A., Tabata Y., Takato T., Nagawa H. (2009). Selective and Sustained Delivery of Basic Fibroblast Growth Factor (BFGF) for Treatment of Peripheral Arterial Disease: Results of a Phase I Trial. Eur. J. Vasc. Endovasc. Surg..

[199] Kusuhara H., Itani Y., Isogai N., Tabata Y. (2011). Randomized Controlled Trial of the Application of Topical B-FGF-Impregnated Gelatin Microspheres to Improve Tissue Survival in Subzone II Fingertip Amputations. J. Hand Surg. Eur. Vol..

[200] Liu J.Y., Hafner J., Dragieva G., Seifert B., Burg G. (2004). Autologous Cultured Keratinocytes on Porcine Gelatin Microbeads Effectively Heal Chronic Venous Leg Ulcers. Wound Repair. Regen..

[201] Liu J.Y., Hafner J., Dragieva G., Burg G. (2004). Bioreactor Microcarrier Cell Culture System (Bio-MCCS) for Large-Scale Production of Autologous Melanocytes. Cell. Transpl..

